# Approaches to Monitor Circuit Disruption after Traumatic Brain Injury: Frontiers in Preclinical Research

**DOI:** 10.3390/ijms21020588

**Published:** 2020-01-16

**Authors:** Gokul Krishna, Joshua A. Beitchman, Caitlin E. Bromberg, Theresa Currier Thomas

**Affiliations:** 1 Barrow Neurological Institute at Phoenix Children’s Hospital, Phoenix, AZ 85016, USA; gokulkrishna@email.arizona.edu (G.K.); joshua.beitchman@gmail.com (J.A.B.); cehair@email.arizona.edu (C.E.B.); 2 Department of Child Health, University of Arizona College of Medicine-Phoenix, Phoenix, AZ 85004, USA; 3 College of Graduate Studies, Midwestern University, Glendale, AZ 85308, USA; 4 Phoenix VA Healthcare System, Phoenix, AZ 85012, USA

**Keywords:** traumatic brain injury, neurotransmitters, circuits, behavior, morbidity, electrochemistry, glutamate, dopamine, post-concussive symptoms, microbiota

## Abstract

Mild traumatic brain injury (TBI) often results in pathophysiological damage that can manifest as both acute and chronic neurological deficits. In an attempt to repair and reconnect disrupted circuits to compensate for loss of afferent and efferent connections, maladaptive circuitry is created and contributes to neurological deficits, including post-concussive symptoms. The TBI-induced pathology physically and metabolically changes the structure and function of neurons associated with behaviorally relevant circuit function. Complex neurological processing is governed, in part, by circuitry mediated by primary and modulatory neurotransmitter systems, where signaling is disrupted acutely and chronically after injury, and therefore serves as a primary target for treatment. Monitoring of neurotransmitter signaling in experimental models with technology empowered with improved temporal and spatial resolution is capable of recording in vivo extracellular neurotransmitter signaling in behaviorally relevant circuits. Here, we review preclinical evidence in TBI literature that implicates the role of neurotransmitter changes mediating circuit function that contributes to neurological deficits in the post-acute and chronic phases and methods developed for in vivo neurochemical monitoring. Coupling TBI models demonstrating chronic behavioral deficits with in vivo technologies capable of real-time monitoring of neurotransmitters provides an innovative approach to directly quantify and characterize neurotransmitter signaling as a universal consequence of TBI and the direct influence of pharmacological approaches on both behavior and signaling.

## 1. Introduction: Acute Chronic Deficits of Mild TBI

Affecting over 2.5 million people across the United States, traumatic brain injury (TBI) is the leading cause of non-fatal injuries [1,2]. Over 75% of TBI patients that report to emergency departments are diagnosed with mild TBI. Mild TBI is defined as an acute brain injury as a result of mechanical energy to the head from external physical forces that cause a brief loss of consciousness and or alteration of mental state (Glasgow coma scale score 13–15 at 30 min after the impact) without loss of tissue [3].

Damage after TBI can be classified as focal or diffuse injury. Focal brain injuries often produce overt injuries such as skull fracture, intracerebral and subdural hematomas, subarachnoid hemorrhage with significant elevations in intracranial pressure [4]. Diffuse TBI is the result of rapid rotational, and/or liner acceleration and deceleration forces cause widespread damage to the neuronal and vascular structures with no overt pathology identified by imaging studies [5]. Studies in humans and animals have shown neuronal pathology, microvascular disruption, white matter injury and axonal disconnection [6,7]. The biomechanical forces primarily cause metabolic dysregulation with imbalances in the ionic flux and disruption of the blood-brain barrier (BBB) that further exacerbate the damage [8,9,10]. The white matter injury resulting from axonal injury has the potential to disconnect neuron-specific relays between brain regions with implications for animal and patient behavioral morbidities. The diffuse axonal injury (DAI) is the earliest pathognomonic feature of mild TBI and is likely the key determinant of the long-term outcome [11]. The pathophysiological cascade involves secondary injuries that gradually evolves over seconds to months after primary injury. The secondary injuries involve metabolic and cellular derangements with neurometabolic cascades of unregulated neurotransmitter release (initiated by mechanical brain tissue deformation), increased oxidative stress, free radical production, inflammation and cell swelling [12,13,14] that exacerbate the primary injury and precipitates behavioral/mental deficits. These acute pathological perturbations are associated with early clinical characteristics of TBI with implications for long-lasting impairments [15]. Importantly, the heterogeneity and diversity in the pathogenesis of diffuse TBI by disrupting brain circuits emphasize the importance of considering the course and morbidity of injury.

Mild TBI cases (20–50%) develop persistent morbidities within one-month post-injury [16]. Patients often suffer transient symptoms that can persist months after initial injury with impaired brain function in cognitive, affective, somatic, and motor domains [17,18]. Neuropsychological tests in patients and neuropathological assessments in experimental TBI indicate continued posttraumatic symptoms that can persist for months to years after TBI [19,20,21,22] or late-onset symptoms of variable in duration with subjective symptomologies [23]. Post-concussive symptoms (PCSs) involve common constellation of symptoms reported by patients after mild-to-moderate TBI. PCSs have been previously diagnosed based on the International Classification of Diseases (ICD)-10, or based Diagnostic and Statistical Manual of Mental Disorders (DSM)-IV criteria as symptoms and problems in three or more multiple domains involving (1) headache, dizziness, general malaise and excessive fatigue, (2) mood and emotional changes such as irritability, depression and/or anxiety, (3) lack of concentration and memory deficits; and (4) fear of permanent brain damage [24]. It is estimated that 5.3 million survivors currently live with PCSs, which contribute to the disability-adjusted life years, impairment of activities in daily living, impede or can complicate return to the work [25,26]. The underlying mechanisms of persisting and emerging PCSs likely arise as a consequence of impaired activation and function of brain circuitry that occurs in response to adaptive or maladaptive compensation. From this perspective, the theme of circuit disruption has emerged as a potential explanation for many of the observed PCSs. The development of PCSs is also influenced by several factors including, but not limited to, personality, mental health, physical health, social status, gender, nutrition, alcohol, and substance use, that also may affect the rate of recovery after injury [27,28,29]. Together, these pre-injury conditions influence a patient’s recovery and prognosis.

Although diffuse white matter disconnections are a characteristic, multicentric outcome of the mechanical impact of TBI, the neurological impairment may reflect dysfunction rather than just neuronal damage. The initial injury and subsequent injury cascade influence the functional state of surviving neurons, astrocytes and microglia and compensatory alterations involving multiple neurotransmitter systems that lead to synaptic deficits that lead to deficits in circuit function. Insights into the neuronal circuits reveal anatomically unique changes in several neurotransmitter systems that modify neuronal dynamics, synaptic function, receptors and transporters [15,30,31]. Our understanding of the development of morbidity and targets for recovery is likely to be informed by studying a variety of neurotransmitter signaling through the application of electrochemical techniques.

## 2. Current Animal Models of TBI

Research into the pathology and pathophysiology of TBI has been rapidly advanced by the development of animal models. So far, the animal models replicate injury types, providing a better understanding of molecular and cellular mechanisms, neurological deficits along with possible rehabilitative/neuroprotective strategies. Rodent and non-human primate TBI models simplify the complex events observed in humans while consistently and robustly reproducing the primary and secondary injuries. In addition, TBI models have provided the opportunity to evaluate for translatable biomarkers that can track TBI progression in humans.

For direct clinical relevance to diffuse TBI, several diffuse models of injury including fluid percussion, weight-drop, and blast injury models have been developed and modified to provide a preclinical platform recapitulating features of human TBI condition. The fluid percussion injury (FPI) imparts a fluid pressure pulse to the intact dura through a craniotomy which can be located laterally or centrally over the brain (for a diffuse or mixed pathology). The pressure pulse is a variable that can be controlled to alter injury severity. Midline (or central) FPI is predominantly used to produce DAI, petechial hemorrhage, and white matter damage in both hemispheres [32]. In relevance to human TBI, the midline FPI model in rodents has been invaluable in studying the longitudinal pathophysiology of diffuse brain injury. The plethora of information derived from the FPI model has shed light on the pathology involving neurophysiology, neuropathology, neuroimaging and behavioral manifestations that are relevant to clinical consequences [33,34,35]. The weight-drop injury model induces impact acceleration with a falling guided weight on the skull, with or without craniotomy. The severity of the injury can be adjusted by changing the mass of the weight and height drop. Besides widespread DAI, the model induces bilateral neuronal damage with the appearance of petechial hemorrhage that closely mimics human TBI [36]. The blast TBI model is simulated using explosives, compressed air, and gas in either blast or shock tubes to mimic the blast injuries commonly observed among military service members. The model employs a compression-driven shock tube to induce blast wave creating a series of effects involving diffuse cerebral edema, hyperemia, and vasospasm [37,38].

Behavioral alterations have been observed in most of the TBI animal models and characterization of behavioral assays is essential for understanding neural systems influencing the behavioral phenotype. As a valuable read-out to probe detailed mechanisms of functions and dysfunctions, various behavioral paradigms help characterize the behavioral repertoire similar to the acute and chronic sequelae that TBI survivors suffer. The assays provide for the sensitive, objective, and quantifiable behavioral measures with the use of video tracking, automated recordings, and detection technologies to record and analyze the behaviors. The assays include neurobehavioral assessment [39], exploratory behavior [40,41], learning and memory [42], and sensory sensitivity [43,44]. These behavioral models have a high degree of face validity, wherein the observable phenotype in the animal reproduces the human TBI condition.

## 3. Circuit Dysfunction after Diffuse TBI: Adaptive and Maladaptive Responses

Animal models have extended clinical findings to show impaired neuroplasticity after diffuse TBI [45]. Neural plasticity is highly adaptive and works to maintain the homeostatic processes by recruiting neurotrophic factors which compensate for the injury-induced adaptations [46,47]. Diffuse TBI initiates a widespread regenerative counter-response that includes reconnection of surviving neurons [48] and collateral sprouting of remaining axons near the denervated regions to create new contacts [47]. Changes in glia and vasculature contribute to the generation of new connections [49,50], where maladaptive reorganization may compound the degree of functional compromise while the injured brain attempts to restore the lost function [51,52]. Yet, it is not well understood the extent to which plasticity is maladaptive and instigates faulty reverberations within circuitry that can have mixed functional outcomes [47]. Indeed, surviving neurons are under metabolic stress and are overwhelmed with increased pathological burden. The ongoing reorganizational processes likely contribute to the observations of late-onset behavioral deficits. Thus, it is reasonable to predict that behavioral morbidities underlying PCSs arise as a consequence of multi-level adjustments that occur over time post-injury as a result of cellular modifications, alterations in neurotransmission and circuit reorganization.

## 4. TBI-Induced Changes in Neurotransmitter Signaling: Acute and Chronic Responses to TBI

Dysregulation of the neurotransmitter systems has been associated with several neurodegenerative diseases as well as neuropsychiatric disorders. Alterations in neurotransmitters after TBI leads to compensatory alterations in neurotransmitter receptors and related signaling pathways, important for circuit-level information transmission. The function of brain circuits is governed, in part, by several neurotransmitter systems that regulate neuronal information flow through exocytosis, uptake and recycling events.

### 4.1. Primary Neurotransmitters

#### 4.1.1. Glutamate

Glutamate is the primary excitatory neurotransmitter and more commonly implicated in TBI neuronal susceptibility to excitotoxic injury [53]. Released into the extracellular space through deformation-induced depolarization, stimulation, or exocytosis of synaptic vesicles, glutamate controls circuit function spanning a wide range of spatio-temporal scales. The clearance of glutamate from the extracellular space is regulated by high-affinity, sodium-dependent excitatory-amino-acid transporters (EAATs) on astrocytes, primarily EAAT2 (GLT-1 in rodents) and EAAT1 (GLAST in rodents) [54,55]. Further, damage to vasculature can cause platelet activation that releases glutamate that can permeate the BBB increasing extracellular glutamate [56,57]. Increased extracellular levels of glutamate binds metabolic glutamate receptors that can mediate neuronal damage through secondary injury processes and exacerbating existing neuronal pathology [58,59].

Several studies demonstrate significant glutamate dysregulation as an instigator of acute pathophysiology in days following TBI. Clinical microdialysis studies demonstrate increased extracellular glutamate levels as early as 24 h post-injury and up to 9 days post-injury [60,61,62]. Focal and diffuse TBI rodent models have demonstrated increased extracellular glutamate levels as early as 1 h post-injury [63,64]. TBI-induced increases in extracellular glutamate alter the localization of glutamate receptors and transporters, further impairing extracellular regulation of glutamate, reducing glutamate buffering capacity and reuptake patterns that modulate synaptic signaling [65]. Prolonged activation of NMDA (N-methyl-D-aspartate) and AMPA (α-amino-3-hydroxy-5-methyl-4-isoxazole propionic acid) receptors can induce neurodegeneration linked to the loss of membrane potential and enhanced cytosolic calcium levels after brain injury [66]. While excess glutamate can exacerbate apoptosis or necrosis in damaged neurons, the ability of astrocytes to rapidly and efficiently clear extracellular glutamate levels might be neuroprotective [54] and the extent of neuronal loss from excitotoxicity remains controversial.

#### 4.1.2. GABA (γ-Aminobutyric Acid)

CABA (γ-aminobutyric acid), the main inhibitory CNS (central nervous system) neurotransmitter, is synthesized in inhibitory neurons from glutamate. While also being vulnerable to structural damage, it is also subject to homeostatic derangement following TBI. GABAergic neurons drive inhibitory elements of brain circuitry that shape circuit function through local and projection neurons. The released GABA acts on ionotropic GABA_A_ and GABA_B_ receptors located on the axonal terminals GABAergic transmission is terminated by clearance from the extracellular space by GABA transporters (GATs) on surrounding neurons and glia. GABA receptors regulate information processing with high physiological relevance through the maintenance of phasic and tonic inhibition, and modulation of neuronal excitability [67]. Experimental data have indicated that TBI can cause reduced expression of GAT at 24 h post-injury [68]. Magnetic resonance spectroscopy (MRI) has shown lower GABA levels in the prefrontal cortex associated with memory deficits in professional boxers repetitive mild TBI [69]. Utilizing this technique, recent work has indicated region and time-specific changes in the GABA showing reduced levels in the cerebral cortex at 72 h and two-week post-mild TBI [70]. In the same region, increased GABA_B_ receptor-mediated inhibition has been associated with loss of synaptic plasticity after sports concussion [71,72]. Acutely following TBI, changes in the GABA_A_ expression alters phasic inhibition important for timing-based signaling leading to overexcitation of neurons and membrane depolarization. However, changes in GABA neurotransmission acutely and chronically offset the balance of excitation and inhibition that underlies several neurological diseases. Recently, electrochemical methods to record near real-time neurotransmission of GABA have been developed and open a wealth of opportunity to understand the role of GABA neurotransmission in the contribution to PCSs [73,74].

Glutamate and GABA are responsible for maintaining the excitatory–inhibitory balance required for circuit function. Post-traumatic epilepsy (PTE), a major long-term consequence of TBI, occurs as a result of a shift in the excitation-inhibition balance of the brain circuitry [53]. Anti-epileptic medications seek to restore the excitatory–inhibitory balance for the successful management of seizures. While PTE is most commonly associated with an imbalance in glutamate and GABA, it is important to consider that the excitatory–inhibitory balance is either directly and/or indirectly implicated with all symptoms associated with TBI as evidenced by the numerous types and distribution of transporters and receptors throughout the brain. Ongoing research is necessary to understand time- and region-dependent disruptions of this balance for improved treatment and management of side effects.

### 4.2. Modulatory Neurotransmitters

#### 4.2.1. Dopamine

Accumulating evidence suggests that the catecholaminergic system is highly vulnerable to the effects of TBI. The monoamine neurotransmitter, dopamine (DA), modulates brain function via widespread DA efferent projections. The majority of DA neuron cell bodies are located in the midbrain (substantia nigra, ventral tegmental area), olfactory bulb, and hypothalamus. Midbrain DA neurons have a long and diffuse projection pattern that exposes DA neurons to shearing forces of injury, oxidative stress, impaired plasticity, and neuroinflammation (reviewed in [75]). The dopamine transporter (DAT) regulates the duration of DA in the extracellular space by clearing released DA through presynaptic reuptake [76]. DA plays an important role in cognitive, motor, emotional, motivational, and neuroendocrine processing from disruptions in the striatum, limbic system and frontal cortex [77,78].

Clinical studies implicate a disruption in DA neurotransmission over time post-injury. A computed tomography imaging study in humans has shown reduced striatal DA transporters (DAT) in the striatum after 142 days post-TBI [79]. This could lead to sustained increases of DA levels in the synaptic cleft that can be oxidized to form a quinone moiety that is capable of damaging cellular macromolecules through oxidative stress [80]. Diffusion MRI studies in moderate-severe brain-injured patients displayed reduced substantia nigra volume, and striatal DAT levels associated with cognitive deficits in information processing speed and executive functions [81], with the potential to influence symptoms associated with attention-deficit/hyperactivity disorder (ADHD), Parkinson’s disease (PD) and Huntington’s disease (HD).

Experimental models likewise suggest substantia nigra damage and remote cortical deficits due to damage of long-range axon projections [82]. The nigrostriatal dopaminergic axons have little to no myelin and are therefore more vulnerable to the shearing forces of TBI [83]. TBI augments DA levels and the rate of DA turnover (i.e., the ratio of DA metabolites to DA) at 1 h post-injury, indicating acute changes DA regulation [84]. Tyrosine hydroxylase (TH) is the rate-limiting enzymatic step for DA synthesis. TH and tissue levels of DA were increased in the prefrontal cortex out to 14 days following controlled cortical impact (focal injury) [85]. Moreover, fast-scan cyclic voltammetry (FSCV) measurements of DA neurotransmission have revealed reduced DA release and reuptake in dorsal striatum after 2 weeks of controlled cortical impact injury [86]. Similar to clinical reports, reduced striatal DAT expression has been reported preclinically after focal TBI [87]. The DA projections are highly branched and contain several neurotransmitter release sites. Midline FPI induced extensive DA axonal pathology in medial forebrain (at 1 day post-injury) and enhanced pro-inflammatory cytokines in the striatum (at 6 h post-injury) that is implicated in reducing DA release [88]. Whole tissue HPLC analysis indicated augmented DA turnover with altered DA utilization in rat brain substantia nigra pars compacta after 28 days post-TBI [82].

Although the cognitive problems associated with TBI is multifactorial, a wide range of reports implicate that the disruption to the dopaminergic (DAergic) neurotransmission as an important factor [89,90,91]. A broad range of cognitive domains commonly affected after TBI including memory, learning, impaired attention including defects in sustained attention, information processing speed, and executive functions such as working memory, problem solving, planning, and impulse control. Importantly, treatment approaches for cognitive enhancement crucially depend on the DAergic level since the synaptic levels of DA are non-linearly associated with cognitive function [92].

#### 4.2.2. Norepinephrine

Norepinephrine (NE), also known as noradrenaline, a catecholamine derived from I-tyrosine, has been implicated in the acute and chronic effects of brain injury [93,94]. Most of the experimental TBI studies report inconsistent alterations in levels of NE [85,95]. Time and region-dependent changes in NE turnover were observed after focal TBI with the contralateral reduction in the cerebral cortex and cerebellum after 6 h followed by a bilateral reduction in the hypothalamus, cerebellum, locus coeruleus and medulla at 24 h post-injury [96,97]. However, findings with whole tissue HPLC analysis show an acute increase in NE turnover (30 min post-focal lesion) in the somatosensory cortex [98] and chronic reduction persisted until 8 weeks post-injury [99]. Specifically, NE has been shown to influence processing speed via its alpha-2A receptor that functions to enhance excitability on target neurons [100,101]. At the higher-order connectivity level, NE modulates intrinsic networks, specifically the cognitive (salience) network, dysfunction of which produces impairments of cognitive control following TBI [102]. Treatment of NE dysregulation is considered with patients diagnosed with ADHD, PTSD, and depression.

#### 4.2.3. Acetylcholine

Acetylcholine (ACh) is a fast-acting neurotransmitter of the cholinergic system that alters neuronal excitability, where changes in signaling have consequences linked to attention, drug abuse and food intake [103]. Clinical studies on the neuropathological, electrophysiological and pharmacological dynamics of ACh have provided evidence for cholinergic dysfunction after TBI [104]. Although clinical reports are scant, initial studies in TBI patients have demonstrated altered cerebrospinal fluid cholinergic function with reduced cholinesterase activity [105,106,107]. Increased levels of basal ACh and reduced activity of choline acetyl transferase (ChAT), a constitutive component of cholinergic nerve terminals have also been identified in cerebrospinal fluid of brain-injured rats [108,109]. Postmortem brain samples from patients who died as a result of head injury showed reduced ChAT activity [110], suggesting a decrease in the activity of cholinergic input. Clinical studies indicate that cholinergic drugs have been successfully prescribed for TBI patients with post-traumatic cognitive deficits [111,112]. Despite limited clinical data available in this regard, these findings suggest deficiencies in ACh innervation and synaptic activity after TBI. Loss of cholinergic neurotransmission is also a correlate of AD severity, and the overlapping pathology may be involved with TBI being a risk factor for AD-related disease progression [113,114].

Animal studies have shown acute effects of FPI on increased hippocampal ACh levels [115]. Phosphoryl [^2^ H_9_] choline infusion measurements revealed that TBI alters ACh turnover in the rat midbrain at 12 min and 4 h after moderate FPI, indicating enhanced cholinergic transmission [116]. Cognitive impairments are the most common long-term deficits after TBI persisting for several years after the injury. Regulation of attention, memory, and executive functions constitute significant components of the cortical cholinergic system that are disrupted after TBI [117,118]. Experimental reports have linked impaired cognition after TBI to chronic changes in cholinergic neurotransmission suggesting reduced ChAT in limbic brain regions [119]. It has been previously demonstrated that controlled cortical impact-induced focal brain injury causes significant reduction in hippocampal high-affinity [^3^H] choline uptake thus suggesting that the cholinergic dysfunction is mainly associated with lower ability of cholinergic neurons to clear extracellular choline [120]. In vivo microdialysis technique has shown muscarinic antagonist evoked lower concentrations of ACh release in hippocampus and neocortex associated with spatial memory deficits among rats after controlled cortical impact brain injury [121,122]. At chronic time-point, post-controlled cortical impact, decreased ACh evoked release was associated with persistent cognitive deficits using microdialysis freely-moving behaving animals [123]. Other findings support that TBI-induced loss of ChAT was associated with cholinergic dysregulation [124]. There is also evidence that the compensatory response to TBI involves alterations in vesicular transporter for ACh (responsible for the concentration of ACh within synaptic vesicles) and mediator of cholinergic signaling showing increased expression of hippocampal vesicular ACh transporter (VAChT) at 4 weeks post-injury [125] and reduced muscarinic autoreceptor subtype 2 (M2) immunoreactivity [126].

#### 4.2.4. Serotonin (5-Hydroxytryptamine, 5-HT)

Serotonin (5-hydroxytryptamine, 5-HT) is involved in the regulation of mood, where dysregulation of 5-HT neurotransmission plays a crucial role in neuropsychiatric disorders in the general population and TBI patients [127]. Moderate FPI in rats have shown to enhance levels of 5-HT and 5-hydroxyindoleacetic acid (5-HIAA) during the first 10 min after TBI [128]. However, the metabolite 5-HIAA extracellular levels were decreased initially during the first 10 min after diffuse TBI. Recent findings reveal that TBI reduces reuptake of 5-HT via modulation of the serotonin transporter (SERT) to prevent replenish of neuronal stores of 5-HT and consequently serotonergic transmission [129]. It has been shown that TBI-induced neuroinflammation decreases serotonin/tryptophan ratio at 21 days post-injury [130]. Overall, preclinical evidence suggests that 5-HT neurotransmission is decreased over time, contributing to the manifestation or worsening of neuropsychological symptoms.

The chronic consequences of TBI on psychiatric health have been identified not only after severe injury but also in several cases classified as mild to moderate. Psychiatric problems can be a major clinical problem as it potentially interferes with rehabilitation and overall recovery. The 5-HT system has wide distribution throughout the brain regulating various behavioral processes [131]. Over one-third of TBI patients develop major depression with increased risk linked to injury severity [132]. Selective serotonin reuptake inhibitors (SSRIs) are currently the first-line treatment for PTSD, depression, anxiety, and other mood disorders following TBI [133]. SSRIs are also used in the management of AD, PD, HD, and epilepsy. However, the relationship between this treatment and TBI is still under consideration.

### 4.3. Conclusion

There is growing evidence that changes in neurotransmission associated with TBI can instigate, accelerate, or exacerbate symptoms associated with chronic traumatic encephalopathy, epilepsy, PD, AD (and other forms of dementia), amyotrophic lateral sclerosis, HD, PTSD, ADHD, anxiety disorders, and depression disorders [134,135]. Modulation of primary and modulatory neurotransmission is primarily targeted for therapy. Yet the etiology of circuit dysregulation is still poorly understood. Progress in diagnosis and treatment of TBI is accounted for by growing acknowledgment that TBI is a neurological disease process rather than a single event that is a risk factor for several neurological disorders and diseases.

## 5. Biosensors for In Vivo Neurotransmitter Monitoring

Studies evaluating the extracellular dynamics of neurotransmitters can provide critical information regarding neuronal communication that is disrupted after TBI. Insight may be gained into aspects of functional neuronal communication by evaluating region-dependent pre-synaptic release, extracellular clearance, basal concentrations in anesthetized animals and behaviorally relevant signaling in freely-moving animals. Several techniques have been developed and applied for in vivo measurement of neurotransmission in various brain disorders. The functional role for neurotransmitter release events in the regulation of homeostasis, as well as influence on behaviors, have been previously reported. The use of cell-based fluorescent sensors [136], microdialysis [137] and fast-scan cyclic voltammetry [138] have been employed to probe changes in neurotransmitter signaling in naïve and models of neurological deficits. However, the development of devices with improved spatial and temporal resolution has helped capture endogenous, micromolar alterations to neurotransmitter release and reuptake.

Electrochemical detection with biosensors allows for real-time observation and quantification of neurotransmitter events. Biosensors often use a biological element (enzyme) to bind a specific analyte of interest and through the application of current, can detect and quantify the extracellular levels and kinetics of release and clearance at 2–100 Hz. The size of the sensors has also been decreased and in some instances, consists of several microsensors on a single electrode. Application of various exclusion layers to the microsensors during the fabrication process allows for increased selectivity, to avoid recording endogenous electroactive molecules that can prevent the detection of the analyte of interest. In vitro calibration allows for calculations where the current generated with applied potential is proportional to the analyte concentration. Electrochemical biosensor design continues to evolve with the recent implementation of thin needle-type probes that improve spatial resolution within subregions and small nuclei. The enzyme-modified electrodes and fast-scanning cyclic voltammetry behave been notably employed for spatial monitoring of rapid neurotransmitter dynamics. Fast-scanning cyclic voltammetry applies a triangular voltage wave to a carbon fiber electrode at high scan rates (300 V s^−1^). The microelectrode measures the current as cyclic voltammogram as a characteristic oxidation and reduction peaks by subtracting the large background current. Other biosensors use enzyme-based microelectrodes with amperometric detection that provides the advantage of better resolution and minimal invasion/tissue damage [139]. The application of constant potential oxidizes or reduces the analyte of interest depending on intrinsic electrochemical properties to detect current that is linear to the electroactive activity in the biological tissue surrounding the microelectrode [140]. While most traditional neurotransmitters follow basic Michaelis-Menten kinetic predictions, non-classical functions can also occur, including spontaneous events, quasi-paracrine action, extrasynaptic overflow, transporter, receptor and channel-mediated release [141,142,143]. These non-classical and subtle changes associated with chemical transmission require novel approaches to data analysis and systematic neuropharmacological challenges to improve associations with behavioral relevance. Albeit, the evolution in technology allows for minimally invasive approaches to directly monitor and manipulate neurochemical transmission that measures fast fluctuating transients (quantity, timing, and dynamics) in spatially discrete relays of behaviorally relevant circuitry.

## 6. Brain-Injured Circuitry and Behavioral Morbidities: The Whisker Barrel Circuit

Diffuse TBI pathology is widespread, resulting in molecular, structural and functional alterations throughout the neuroanatomy that results in disrupted circuit function. At the basis of the pathology are the primary and secondary insults to neural circuits that determine the observed behavioral output. Thus, evaluation of these their anatomical connections is essential to understand how TBI-induced pathology alters functionality. One example of this is highlighted in the somatosensory thalamocortical circuit of the whisker barrel circuit (WBC) in rats. The somatosensory WBC in rodents processes sensory information from the mystacial whiskers for assessing spatial and textural information through thalamocortical and corticothalamic circuitry [144]. Whisker somatosensation represents the primary sensory modality of rodents and is represented by a large portion of the brain for processing, similar to that of touch, sight, and hearing in humans. Each whisker is primed for sensory integration, consisting of large follicles with multiple superficial and deep vibrissal nerves associated with somatotopically distinct trigeminal neuron projections [145]. Trigeminal neurons project to the primary sensory nucleus (PrV), to the barreloids of ventral posterior medial (VPM) nucleus of the thalamus, to the primary somatosensory cortex forming discrete clusters in layer 4 (S1BF), which form the basis of the ‘barrel’ fields [146,147,148,149,150]. The somatotopographical localization of each whisker makes the WBC valuable to study circuit reorganization as evidenced by its history of use to study neuroplasticity in response to peripheral nerve injury, developmental patterning, neural coding, and neural computation [151,152,153]. As rodents largely rely on whisker function for environmental exploration due to poor eyesight, this circuit provides an ideal model to assess TBI-induced longitudinal circuit disruption followed by compensatory events that manifest in behavioral morbidity.

As observed by Thomas et al., TBI-induced pathology in the VPM and S1BF results in maladaptive circuit regeneration resulting in the development of late-onset, persistent sensory sensitivity to whisker stimulation during the whisker nuisance task (WNT) [44,154,155]. The WNT evaluates the behavioral response of the rat to tactile stimulation through three consecutive 5 min bouts of manual whisker stimulation. Naïve and uninjured sham rats are ambivalent to the stimulation, whereas TBI results in increased avoidance, evasiveness, and aggressiveness that is significant by 4 weeks and maintained to 8 weeks post-injury [154]. Hypersensitivity to whisker stimulation is exacerbated by elevated intracranial pressure (ICP) following FPI concurrent with neuronal somatic membrane damage and poration [156,157]. The WNT has been a reproducible approach to measuring circuit abnormalities across laboratories [154], injury models [158], and rodent species [159]. TBI-induced sensory hypersensitivity observed in the WNT can be likened to visual and auditory hypersensitivity commonly experienced by brain injury survivors [160,161], serving as an excellent in vivo model to longitudinally assess how maladaptive circuit reorganization after TBI leads to late-onset behavior morbidity.

The use of whisker barrel circuit serves as an ideal model to study TBI-induced neurotransmission in a behaviorally relevant circuit, showing hypersensitive glutamate signaling to be associated with the severity of response to the WNT [44]. Electrochemistry using glutamate selective MEAs were used to record real-time potassium (KCl)-evoked glutamate release within relays of the whisker barrel circuit (a glutamatergic circuit) after moderate severity FPI in anesthetized animals [162,163]. Local application isotonic KCl solution depolarized synaptic terminals near the microelectrode, evoking the release of glutamate. Evoked glutamate concentrations in the S1BF and VPM thalamus was significantly increased by 28 days post-injury, with robust responses in the dorsal and medial VPM. Glutamate clearance kinetics and glutamate transporter gene expression were similar between sham and 28 day FPI rats, indicating that increased KCl-evoked glutamate release was not a result of slower glutamate clearance from the extracellular space. Furthermore, KCl-evoked release in injured animals was more sensitive to ω-conotoxin, a voltage-gated calcium channel inhibitor, indicating that increases in KCl-evoked glutamate release were presynaptic in origin. VPM (and S1BF) responses correlate with the severity of sensory hypersensitivity evaluated by the WNT, indicating that changes in circuit function are impacting behavioral responses [44]. This study highlights how in vivo electrochemistry can be used to isolate aspects of neurotransmission relevant to behavioral morbidity, but it is also limited by evaluation in an anesthetized animal. While a presynaptic mechanism is implied, replicating these studies in awake behaving rats can provide behaviorally induced glutamate signaling that will provide additional information regarding how the signaling changes (increased bouts of released glutamate, increase concentrations, oscillatory signaling, etc).

Previously, studies on the contribution of circuit dysfunction to post-traumatic morbidity, in the form of neurotransmission, have relied on measurements obtained by microdialysis techniques. Microdialysis involves implantation of a probe with a 1–2 mm (for rats and mice) semi-permeable membrane for diffusion of extracellular neurotransmitters in artificial cerebral spinal fluid that is collected and assessed using HPLC. The probe and cannula implantation cause tissue damage 300–500 µm away for the probe [164]. Neurotransmitters are typically tightly regulated producing short fluxes of extracellular concentrations. Each sample is collected every 1–10 min, which does not accommodate the detection of the rapid release and uptake of many neurotransmitters [165,166]. The larger diameter (150 to 400 um) of the dialysis probe and constant infusion with artificial CSF can result in neurotransmitter dilution in the sampled region [167]. Microdialysis provides information on multiple neurotransmitters and their metabolites, peptides, and hormones in freely-moving animals, having many advantages along with these disadvantages highlighted here (reviewed in [168]). Recent advances using the genetically encoded neural activity indicators and two-photon imaging methods provide for non-invasive visualization of neurotransmitter release and neuronal activity [169,170]. Further, the microdialysis technique has been used in human patients to assess the amino acid concentrations in the brain following trauma [171]. However, these techniques lack information on the quantity of neurotransmitters in the extracellular space, particularly the dynamics of neurotransmitter clearance that shape the amplitude and duration of postsynaptic responses. Electrochemical biosensors have addressed temporal dynamics, improved spatial resolution, decreased tissue damage (in comparison with microdialysis), and the ability to evaluate near real-time dynamics of neurotransmitter release and clearance, while sacrificing the ability to evaluate multiple targets [137,172,173].

## 7. Electrochemical Biosensors for In Vivo Monitoring of Neurochemical Signaling

During the decades that neurochemical measurements largely relied on microdialysis, parallel developments were made on methods to measure at second and sub-second timescale with improved resolution. The developments were necessary since the cascade of events that follow signal transduction in neurotransmitter signaling involves a vast range of spatial and temporal scales. Initially, the microelectrodes developed were carbon-fiber based with low reproducibility and not suitable to measure molecules that could not be oxidized or reduced, like glutamate, GABA and ACh. Progress in technical advances over the past several years in microfabrication methods to create electrochemical biosensors containing more than a single microelectrode (microelectrode arrays; MEAs) have increasingly shown to be effective tools for neurotransmission detection. MEAs have served as an interface to develop amperometric enzyme-based biosensors for measurements of neurotransmitters, including electroactive and non-electroactive neurotransmitters in the extracellular space of the brain [174]. There are several unique types of MEAs currently being used to assess neurochemical signaling—varying in size, number of arrays, material, etc. [175,176,177]. Incorporation of multiple sensors allows for improved spatial resolution (in microns) and minimal tissue damage to the surrounding parenchyma [178,179]. The enzymatic biosensors have found wide applicability in brain research with amperometric biosensor recordings. The enzyme-based microelectrode arrays offer a better utility tool to monitor chemical signaling, they overcome the limitations associated with low temporal resolution and provide continuous in vivo monitoring at higher sampling rates. Their low limit of detection provides for near real-time measurement of chemical signaling involving synaptic events and neurotransmitter overflow. MEA coupled with amperometry has been employed to measure glutamate, GABA [74], adenosine [180], ACh [181], lactate [182] and glucose [183]. The non-electroactive neurochemicals are detected by immobilizing oxidase enzymes in a suitable polymeric film onto the electrode surface combined with the amperometric detection of hydrogen peroxide as a reporter molecule. Platinum or its alloys are commonly used as electrode recording surface materials due to their excellent electrocatalytic activity [184]. Importantly, in vivo biosensing has been achieved by polymer modification by enzyme immobilization using glutaraldehyde in the presence of bovine serum albumin onto the surface of microelectrodes [185]. The MEA has been employed to monitor transient changes in extracellular glutamate in animal models of Alzheimer’s disease [186], epilepsy [187], stress [188], aging [189] and TBI [44,162,190]. Miniaturization of MEAs enables stereotaxical lowering into discrete anatomical regions of interest and allows long-term neurotransmitter recordings. A limitation of MEAs is that they can lose sensitivity due to loss of an exclusion layer. Yet detection of this loss is immediate and therefore does not impede interpretation of the data. Depending on the design of the MEA, they are also restricted in the number of extracellular analytes they can measure in comparison to microdialysis. MEAs report current variations shaped by several variables including proper preparation and prior calibration for successful use. The measurement of interferents and metabolites can potentially be overcome by surface modification of MEA as well as by using self-referencing techniques. Future technological improvements in the design and fabrication of implantable electrodes are needed to resolve these limitations.

## 8. Moving into the Future: Recordings in Freely-Moving Animals

In vivo electrochemical recordings using amperometry in awake, freely moving animals allow for assessment of real-time neurotransmitter changes during behavioral tasks. This remained challenging due to the depth of the brain regions and the necessity to record from freely behaving animals, but advances in microelectrode fabrication allow for probing chemical events for longer-term experiments. Several studies have begun to use this approach to study spontaneous neurochemical events in relation to well-defined, quantifiable behaviors of feeding, reward-seeking, sleep-wake, addictive and attention-deficit hyperactivity behaviors [191,192,193,194,195]. Continuing to study the dynamic neurotransmitter changes and behaviors in other disease models including TBI would benefit greatly to our understanding of post-traumatic neuronal signaling. These recordings can also be synched to video recordings of behavior, linking behavior to amperometric recordings. Direct assessment of neurotransmission provides for simultaneous measurement with other measurements of neuronal activity with millisecond precision. Emerging approaches involve the design of microelectrodes capable of concurrent assessment of neural electrical activity with local field potentials (LFPs) [196,197]. The integration of neurochemical information with electrophysiological approaches will greatly expand our understanding of the circuit-neurotransmitter relationship. Moreover, the integrative approach has been validated by several studies [198] providing key insight into both normal neural circuit dynamics and its dysregulation in disease [199].

Advances in in vivo assessments have enabled us to gain insight into the nature of the subthreshold events that underlie evoked action potential discharge in anesthetized and awake animals. Specifically, direct measurement of brain chemical signaling provides valuable information when combined with other physiological methods to manipulate neuronal activity with sophisticated observational work and casual manipulation studies. Targeting neuronal systems infers causality between neurotransmitter events and behavior in conjunction with optogenetic-based [197] or chemogenetic (DREADD) approaches [200]. Optogenetics combined with in vivo electrochemical assessments has already proven to be effective in studying glutamate signaling in both hippocampus and frontal cortex in anesthetized animals [197,201]. This is particularly important for the study of circuit-level mechanisms of disease in vivo, when applied to existing behavioral models as output measures can provide insights for future clinical translation. Changes in metabolic and vascular response occurs as a consequence of neuronal activity to restore brain homeostasis, altering tissue oxygenation. Such changes form the basis of neuroimaging methods such as functional magnetic resonance imaging. Metabolic brain disorders alter oxygen utilization and tissue oxygen tension (*p*O_2_) thereby disrupting neuronal circuits [202,203]. While non-invasive neuroimaging methods provides for a great clinical advantage, the responses observed are influenced from several other factors including hemodynamic response and limited spatial resolution [204]. The ceramic-based platinum MEAs have been used for in vivo recording of changes in *p*O_2_ in anesthetized rat brain with excellent electrocatalytic activity towards oxygen reduction and low detection limits [177]. In a proof-of-concept experiment, our lab recently demonstrated robust reduced oxygen consumption and decreased LFPs in the whisker barrel circuit after mFPI at 1 day post-injury [205], implicating alternative approaches for monitoring changes in behaviorally relevant circuit function after TBI.

Exceptional progress had been made towards electrochemical analysis of behaviorally relevant neurotransmitter signaling in the extracellular space as demonstrated by recordings in non-human primates [206,207] and swine [208]. In 2013, a new wireless sensing device coupled with voltammetric detection for electrochemical monitoring was demonstrated in a late-stage PD [209]. An emphasis has been made towards further miniaturizing biosensors to record at the level of the synapse, increasing temporal resolution for more accurate changes in release/reuptake, improving selectivity (distinguish neurotransmitters) and signal-to-noise, and increasing the number of targets that can be simultaneously recorded. Several strategies have also been developed to minimize tissue damage during insertion, biocompatibility, attenuate foreign body response following implantation, increase longevity, improve performance and resolution, reduce tethering artifact (i.e., wireless), and increase electrode flexibility by multiple groups. However, these approaches have yet to be combined into a single MEA [210]. While limitations to clinical applications still exist, the evolution of this technology is rapidly moving toward a point where the benefit of implantation and the information gained from recordings will outweigh the risk to the patient.

## 9. Peripheral Influences on Neurotransmission and PCSs

Throughout this review, we have focused on circuit reorganization and dysfunction as primary mechanisms towards changes in neurotransmitter signaling after TBI. The following section is intended to acknowledge peripheral influences that have recently been indicated in mediating neurotransmission that may be relevant to treatment and application for in vivo neurotransmitter monitoring.

### 9.1. Neuroendocrine and Neuroimmune Interactions

Emerging studies have reported that a substantial population of TBI patients, as high as 25%, report chronic endocrine dysregulation involving growth hormone, gonadal, thyroid, adrenal and antidiuretic hormones [211,212,213]. Hormone receptors are located on neurons and can influence circuit function. Yet, few studies address the impact of neuroendocrine dysregulation on neurochemical signaling related to TBI-induced PCSs exists. For example, after experimental TBI, chronic dysregulation of the HPA-axis has been implicated by changes in baseline circulating glucocorticoid levels and in response to stress in a sex-specific manner [214,215,216]. Glucocorticoid receptors are located on every cell type and modulate neurotransmission and neuroinflammatory response [217]. With the growing recognition of neuroendocrine–immune interactions, the potential microglial contributions to endocrine dysregulation have been proposed based on the reactivity state of the microglia [218]. The enhanced neuroinflammatory response stemming from immune-mediated central glia, particularly the microglial response might set the stage for depressive-like features [219].

### 9.2. Sex Hormones

Sex-related differences in human brain structure and susceptibility to neuropsychiatric diseases have been documented [220]. Yet, again, their role in the persistence and development of PCSs have not been fully explored. Reports indicate sex differences in many neurotransmitters including serotonergic, cholinergic and adrenergic systems [221,222,223]. Findings also suggest that sex hormones, including estrogen and progesterone, mediate worse outcomes among women of childbearing age after TBI [224]. Ovarian hormones are known to exhibit modulatory role on synaptic transmission regulating presynaptic neurotransmitter release or postsynaptic receptor action as a possible target for their influence after TBI [225,226].

### 9.3. Gut–Brain Axis

There has been a growing support of the influence of multiorgan responses on the acute and chronic effects of TBI. Such interactions between the brain and the systemic physiology involving the function of body organs after TBI can indirectly influence neurotransmitter systems or modulators. The sequela of TBI induces significant alterations in metabolic and enteric function, affecting peripheral systems. TBI survivors often report of gastrointestinal dysfunction with reports increased gut permeability [227], reduced gastric emptying [228] and intestinal contractility [229]. Two communication routes for the gut–brain axis are through the vagus nerve and the gut microbiota [230]. Vagus nerve stimulation is currently being evaluated preclinically and clinically for improvement in motor, cognition, edema, inflammation, and BBB breakdown [231]. Gastrointestinal disorders consequently disrupt the microbiota (gut bacteria) and accumulating data suggesting that the microbiome is the key regulator of the bidirectional gut–brain axis to influence brain function, behavior and host physiology [232]. Preclinical reports have shown microbiota dysbiosis after TBI [233,234]. Yet the link to PCSs has not studied. Evidence points to behaviors linked to neurotransmission that are influenced by microbiota [235] with certain bacteria possessing the capacity to generate neurotransmitters and/or precursors to neurotransmitters. It has been found that several bacteria including *Bifidobacterium* and *Lactobacillus* spp. produce GABA, *Bacillus* spp. produce DA, *Lactobacillus* spp. produce ACh and *Escherichia* spp. produce 5-HT [236,237,238,239] while their implications on brain function are slowly being unraveled. Particularly, the gut microbiome is known to regulate tryptophan metabolism necessary for the synthesis of 5-HT in the brain [240]. The intestine can also serve to be an endocrine organ through the production of microbial neurometabolites. Specifically, GABA is produced directly or indirectly by certain commensal microbes to influence gut–brain interactions [241].

### 9.4. Other Organs

Mild TBI enhances liver inflammatory markers by altering the redox homeostasis and given their wide implications for their interaction between brain and body, leakage of pro-inflammatory factors through the disrupted BBB can exacerbate brain injury pathology [242,243,244]. TBI can promote cardiovascular risk factors through increase in expression of pro-inflammatory chemokines and apoptosis [245,246]. The spleen is an important lymphoid organ innervated by sympathetic nerve fibers that are in contact with splenic immune cells to create a neuroimmune link [247]. Enhanced pro-inflammatory levels have been characterized as an acute response to diffuse TBI [248]. The neurotransmitters of the sympathetic nervous system bind to receptors on the surface of immune cells and within the microenvironmental niche of the spleen, immune cell receptors bind neurotransmitters to exert effects on nerve terminals [249].

## 10. Concluding Remarks

**Figure 1:**
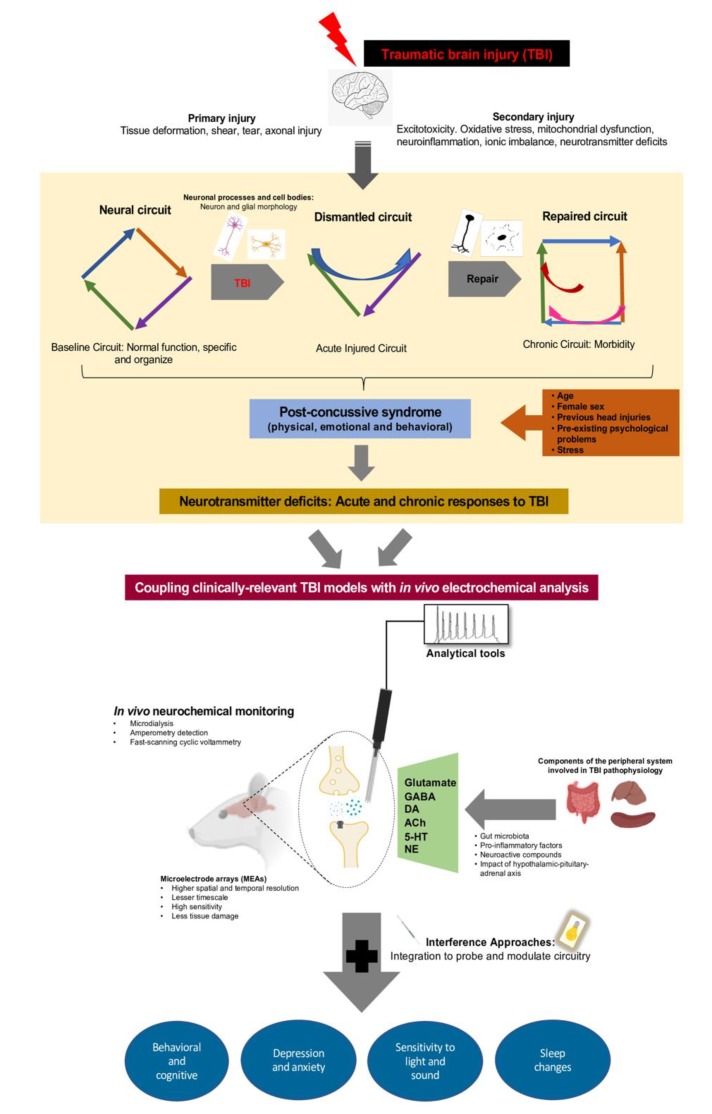
Cartoon summary highlighting the approaches to monitor circuit disruption and behavioral manifestations after traumatic brain injury (TBI). Mild TBI initially manifests as primary and secondary injuries leading to acute and chronic neurological deficits, contributing to morbidity of injury. The repair and reconnection of broken circuitry that follows TBI leads to formation of maladaptive circuitry. The characteristic pattern following injury provides potential context for pathologies of neuronal processes and cell bodies subsequent to injury-related deficits in metabolism or neuronal function. TBI-induced damage to neural responses evolve into diffuse circuit disruption leading to development of post-concussive symptoms (PCSs). Neurotransmitter systems are important components of the neuronal circuitry (also influenced by components of peripheral system) that modulate many of the behavioral functions that are impaired following TBI. The assessments of these neurotransmitter changes can capture important aspects of brain-injured circuitry and offers a potential target for modulation. Experimental studies involving use of different methods for recording extracellular neurotransmitter levels provides for evaluating changes in neurotransmitter signaling, where measurements are made with high spatial and temporal resolution. Coupling clinically-relevant TBI models that show chronic behavioral deficits with in vivo technologies capable of real-time monitoring of neurotransmitters in behaviorally relevant circuitry provides a powerful and innovative approach to understanding compensatory changes in neurotransmitter signaling as a TBI consequence. In experimental models, the use of interference tools permits more direct modulation of in vivo control of neurotransmitter signaling in animals engaged in freely moving and defined behaviors. This approach can be used to understand the impact of pharmacological interventions on both therapeutic regulation of TBI altered neurotransmission capable of mitigating behavior.

In this review, we have discussed the clinical and experimental evidence on the acute and chronic pathophysiological responses after mild TBI, animal models of diffuse brain injury, alterations of neurotransmitter systems that underlie the circuit deficits which can compromise and/or compensate the processes that enables neuronal response to injury. Alterations in the TBI-induced neurotransmitter systems that form the core machinery expressed by most of the neurons have been considered crucial in the development of PCSs. Multiple in vivo electrochemical measurements with increasing resolution offers a portal to directly quantify and characterize the dynamic real-time neurotransmitter signaling mechanisms that operate during behavior to provide important details of the pathophysiology of circuit function. Advances in the application of interference tools permit more direct modulation of in vivo control of neurotransmitter signaling in animals engaged in freely moving and defined behaviors (see Figure 1 for details). Given the unmet clinical need to modulate neurochemical signaling in the diseased state, including TBI, the future of electrochemical characterization can be fully adapted to apply behavioral shaping pharmacological interventions that normalize neurotransmitter signaling.

## References

[1] Langlois J.A., Rutland-Brown W., Wald M.M. (2006). The epidemiology and impact of traumatic brain injury: A brief overview. J. Head Trauma Rehabil..

[2] Peterson A.B., Xu L., Daugherty J., Breiding M.J. (2019). Surveillance Report of Traumatic Brain Injury-Related Emergency Department Visits, Hospitalizations, and Deaths, United States, 2014. Centers for Disease Control and Prevention, Department of Health and Human Services. https://www.google.com.hk/search?newwindow=1&safe=strict&source=hp&ei=sSocXpaxAY6JoASz0ZnACA&q=Surveillance+report+of+traumatic+brain+injury-related+emergency+department+visits%2C+hospitalizations%2C+and+deaths%2C+United+States%2C+2014.+Centers+for+Disease+Control+and+Prevention%2C+Department+of+Health+and+Human+Services&oq=Surveillance+report+of+traumatic+brain+injury-related+emergency+department+visits%2C+hospitalizations%2C+and+deaths%2C+United+States%2C+2014.+Centers+for+Disease+Control+and+Prevention%2C+Department+of+Health+and+Human+Services&gs_l=psy-ab.3...403.403..1296...0.0..0.0.0.......0....2j1..gws-wiz.cFcNrTBM2Yc&ved=0ahUKEwjWza7TlIDnAhWOBIgKHbNoBogQ4dUDCAU&uact=5.

[3] Zetterberg H., Blennow K. (2016). Fluid biomarkers for mild traumatic brain injury and related conditions. Nat. Rev. Neurol..

[4] Sharp D.J., Fleminger S., Powell J. (2016). Traumatic Brain Injury.

[5] Smith K. (2012). Traumatic brain injury: CT scan does not predict outcome of mild traumatic brain injury. Nat. Rev. Neurol..

[6] Povlishock J.T., Christman C.W. (1995). The pathobiology of traumatically induced axonal injury in animals and humans: A review of current thoughts. J. Neurotrauma.

[7] Farkas O., Povlishock J.T. (2007). Cellular and subcellular change evoked by diffuse traumatic brain injury: A complex web of change extending far beyond focal damage. Prog. Brain Res..

[8] Chodobski A., Zink B.J., Szmydynger-Chodobska J. (2011). Blood–brain barrier pathophysiology in traumatic brain injury. Transl. Stroke Res..

[9] Johnson V.E., Stewart W., Smith D.H. (2013). Axonal pathology in traumatic brain injury. Exp. Neurol..

[10] King A.I. (2000). Fundamentals of impact biomechanics: Part I-biomechanics of the head, neck, and thorax. Annu. Rev. Biomed. Eng..

[11] Medana I.M., Esiri M.M. (2003). Axonal damage: A key predictor of outcome in human CNS diseases. Brain.

[12] Hayes R.L., Jenkins L.W., Lyeth B.G. (1992). Neurotransmitter-mediated mechanisms of traumatic brain injury: Acetylcholine and excitatory amino acids. J. Neurotrauma.

[13] Giza C.C., Hovda D.A. (2001). The neurometabolic cascade of concussion. J. Athl. Train..

[14] Giza C.C., Hovda D.A. (2014). The new neurometabolic cascade of concussion. Neurosurgery.

[15] Blennow K., Brody D.L., Kochanek P.M., Levin H., McKee A., Ribbers G.M., Yaffe K., Zetterberg H. (2016). Traumatic brain injuries. Nat. Rev. Dis. Primers.

[16] Langlois J.A., Rutland-Brown W., Thomas K.E. (2006). Traumatic brain injury in the United States; emergency department visits, hospitalizations, and deaths: Centers for Disease Control and Prevention, National Center for Injury Prevention and Control. https://stacks.cdc.gov/view/cdc/5571.

[17] Centre Task Force on Mild Traumatic Brain Injury (2004). Methodological issues and research recommendations for mild traumatic brain injury: The WHO collaborating centre task force on mild traumatic brain injury. J. Rehabil. Med..

[18] Donovan J., Cancelliere C., Cassidy J.D. (2014). Summary of the findings of the International collaboration on mild traumatic brain injury prognosis. Chiropr. Man. Ther..

[19] Harmon K.G., Drezner J., Gammons M., Guskiewicz K., Halstead M., Herring S., Kutcher J., Pana A., Putukian M., Roberts W. (2013). American Medical Society for Sports Medicine position statement: Concussion in sport. Clin. J. Sport Med..

[20] Hall K.D., Lifshitz J. (2010). Diffuse traumatic brain injury initially attenuates and later expands activation of the rat somatosensory whisker circuit concomitant with neuroplastic responses. Brain Res..

[21] Hou R., Moss-Morris R., Peveler R., Mogg K., Bradley B.P., Belli A. (2012). When a minor head injury results in enduring symptoms: A prospective investigation of risk factors for postconcussional syndrome after mild traumatic brain injury. J. Neurol. Neurosurg. Psychiatry.

[22] King N.S., Kirwilliam S. (2011). Permanent post-concussion symptoms after mild head injury. Brain Inj..

[23] Binder L.M. (1986). Persisting symptoms after mild head injury: A review of the postconcussive syndrome. J. Clin. Exp. Neuropsychol..

[24] Polinder S., Cnossen M.C., Real R.G., Covic A., Gorbunova A., Voormolen D.C., Master C.L., Haagsma J., Diaz-Arrastia R., van Steinbuechel N. (2018). A multidimensional approach to post-concussion symptoms in mild traumatic brain injury: A focused review. Front. Neurol..

[25] Selassie A.W., Zaloshnja E., Langlois J.A., Miller T., Jones P., Steiner C. (2008). Incidence of long-term disability following traumatic brain injury hospitalization, United States, 2003. J. Head Trauma Rehabil..

[26] Chauhan N.B. (2014). Chronic neurodegenerative consequences of traumatic brain injury. Restor. Neurol. Neurosci..

[27] Silverberg N.D., Iverson G.L. (2011). Etiology of the post-concussion syndrome: Physiogenesis and psychogenesis revisited. NeuroRehabilitation.

[28] Thomas T.C., Colburn T.A., Korp K., Khodadad A., Lifshitz J. (2015). Translational considerations for behavioral impairment and rehabilitation strategies after diffuse traumatic brain injury. Brain Neurotrauma: Molecular, Neuropsychological, and Rehabilitation Aspects.

[29] Patricia B., O’Neil D.A., LaPorte M.J., Cheng J.P., Beitchman J.A., Thomas T.C., Bondi C.O., Kline A.E. (2018). Elucidating opportunities and pitfalls in the treatment of experimental traumatic brain injury to optimize and facilitate clinical translation. Neurosci. Biobehav. Rev..

[30] Barkhoudarian G., Hovda D.A., Giza C.C. (2011). The molecular pathophysiology of concussive brain injury. Clin. Sports Med..

[31] Bargmann C.I. (2012). Beyond the connectome: How neuromodulators shape neural circuits. Bioessays.

[32] Rowe R.K., Harrison J.L., Ellis T.W., Adelson P.D., Lifshitz J. (2018). Midline (central) fluid percussion model of traumatic brain injury in pediatric and adolescent rats. J. Neurosurg. Pediatr..

[33] Lifshitz J. (2009). Fluid percussion injury model. Animal Models of Acute Neurological Injuries.

[34] Spain A., Daumas S., Lifshitz J., Rhodes J., Andrews P.J., Horsburgh K., Fowler J.H. (2010). Mild fluid percussion injury in mice produces evolving selective axonal pathology and cognitive deficits relevant to human brain injury. J. Neurotrauma.

[35] Lifshitz J., Rowe R.K., Griffiths D.R., Evilsizor M.N., Thomas T.C., Adelson P.D., McIntosh T.K. (2016). Clinical relevance of midline fluid percussion brain injury: Acute deficits, chronic morbidities and the utility of biomarkers. Brain Inj..

[36] Kilbourne M., Kuehn R., Tosun C., Caridi J., Keledjian K., Bochicchio G., Scalea T., Gerzanich V., Simard J.M. (2009). Novel model of frontal impact closed head injury in the rat. J. Neurotrauma.

[37] Risling M., Davidsson J. (2012). Experimental animal models for studies on the mechanisms of blast-induced neurotrauma. Front. Neurol..

[38] Cernak I., Noble-Haeusslein L.J. (2010). Traumatic brain injury: An overview of pathobiology with emphasis on military populations. J. Cereb. Blood Flow Metab..

[39] Mattiasson G.J., Philips M.F., Tomasevic G., Johansson B.B., Wieloch T., McIntosh T.K. (2000). The rotating pole test: Evaluation of its effectiveness in assessing functional motor deficits following experimental head injury in the rat. J. Neurosci. Methods.

[40] Beitchman J.A., Griffiths D.R., Hur Y., Ogle S.B., Hair C.E., Morrison H.W., Lifshitz J., Adelson P.D., Thomas T.C. (2019). Experimental traumatic brain injury induces chronic glutamatergic dysfunction in amygdala circuitry known to regulate anxiety-like behavior. Front. Neurosci..

[41] Herrera J.J., Bockhorst K., Kondraganti S., Stertz L., Quevedo J., Narayana P.A. (2017). Acute white matter tract damage after frontal mild traumatic brain injury. J. Neurotrauma.

[42] Wu A., Ying Z., Gomez-Pinilla F. (2010). Vitamin E protects against oxidative damage and learning disability after mild traumatic brain injury in rats. Neurorehabil. Neural Repair.

[43] Learoyd A.E., Lifshitz J. (2012). Comparison of rat sensory behavioral tasks to detect somatosensory morbidity after diffuse brain-injury. Behav. Brain Res..

[44] Thomas T.C., Hinzman J.M., Gerhardt G.A., Lifshitz J. (2012). Hypersensitive glutamate signaling correlates with the development of late-onset behavioral morbidity in diffuse brain-injured circuitry. J. Neurotrauma.

[45] Krishna G., Ying Z., Gomez-Pinilla F. (2019). Blueberry supplementation mitigates altered brain plasticity and behaviour after traumatic brain injury in rats. Mol. Nutr. Food Res..

[46] Cramer S.C., Sur M., Dobkin B.H., O’brien C., Sanger T.D., Trojanowski J.Q., Rumsey J.M., Hicks R., Cameron J., Chen D. (2011). Harnessing neuroplasticity for clinical applications. Brain.

[47] Wieloch T., Nikolich K. (2006). Mechanisms of neural plasticity following brain injury. Curr. Opin. Neurobiol..

[48] Povlishock J.T., Katz D.I. (2005). Update of neuropathology and neurological recovery after traumatic brain injury. J. Head Trauma Rehabil..

[49] Cheng H.W., Rafols J.A., Goshgarian H.G., Anavi Y., Tong J., McNeill T.H. (1997). Differential spine loss and regrowth of striatal neurons following multiple forms of deafferentation: A Golgi study. Exp. Neurol..

[50] Xing C., Hayakawa K., Lok J., Arai K., Lo E.H. (2012). Injury and repair in the neurovascular unit. Neurol. Res..

[51] Emery D.L., Royo N.C., Fischer I., Saatman K.E., McIntosh T.K. (2003). Plasticity following injury to the adult central nervous system: Is recapitulation of a developmental state worth promoting?. J. Neurotrauma.

[52] Giza C.C., Prins M.L. (2006). Is being plastic fantastic? Mechanisms of altered plasticity after developmental traumatic brain injury. Dev. Neurosci..

[53] Guerriero R.M., Giza C.C., Rotenberg A. (2015). Glutamate and GABA imbalance following traumatic brain injury. Curr. Neurol. Neurosci. Rep..

[54] Anderson C.M., Swanson R.A. (2000). Astrocyte glutamate transport: Review of properties, regulation, and physiological functions. Glia.

[55] Danbolt N.C. (2001). Glutamate uptake. Prog. Neurobiol..

[56] Schmidt R.H., Grady M.S. (1993). Regional patterns of blood–brain barrier breakdown following central and lateral fluid percussion injury in rodents. J. Neurotrauma.

[57] Bell J.D., Thomas T.C., Lass E., Ai J., Wan H., Lifshitz J., Baker A.J., Macdonald R.L. (2014). Platelet-mediated changes to neuronal glutamate receptor expression at sites of microthrombosis following experimental subarachnoid hemorrhage. J. Neurosurg..

[58] Faden A.I., Demediuk P., Panter S.S., Vink R. (1989). The role of excitatory amino acids and NMDA receptors in traumatic brain injury. Science.

[59] Hall E.D., Andrus P.K., Yonkers P.A., Smith S.L., Zhang J.R., Taylor B.M., Sun F.F. (1994). Generation and detection of hydroxyl radical following experimental head injury. Ann. N. Y. Acad. Sci..

[60] Hillered L., Persson L., Carlson H., Ungerstedt U., Ronne-Engström E., Nilsson P. (1992). Studies on excitatory amino acid receptor-linked brain disorders in rat and man using in vivo microdialysis. Clin. Neuropharmacol..

[61] Vespa P., Prins M., Ronne-Engstrom E., Caron M., Shalmon E., Hovda D.A., Martin N.A., Becker D.P. (1998). Increase in extracellular glutamate caused by reduced cerebral perfusion pressure and seizures after human traumatic brain injury: A microdialysis study. J. Neurosurg..

[62] Chamoun R., Suki D., Gopinath S.P., Goodman J.C., Robertson C. (2010). Role of extracellular glutamate measured by cerebral microdialysis in severe traumatic brain injury. J. Neurosurg..

[63] Ojo J.O., Mouzon B., Algamal M., Leary P., Lynch C., Abdullah L., Evans J., Mullan M., Bachmeier C., Stewart W. (2016). Chronic repetitive mild traumatic brain injury results in reduced cerebral blood flow, axonal injury, gliosis, and increased t-tau and tau oligomers. J. Neuropathol. Exp. Neurol..

[64] Katayama Y., Becker D.P., Tamura T., Hovda D.A. (1990). Massive increases in extracellular potassium and the indiscriminate release of glutamate following concussive brain injury. J. Neurosurg..

[65] Levenson J., Weeber E., Selcher J.C., Kategaya L.S., Sweatt J.D., Eskin A. (2002). Long-term potentiation and contextual fear conditioning increase neuronal glutamate uptake. Nat. Neurosci..

[66] Zipfel G.J., Babcock D.J., Lee J.M., Choi D.W. (2000). Neuronal apoptosis after CNS injury: The roles of glutamate and calcium. J. Neurotrauma.

[67] Farrant M., Nusser Z. (2005). Variations on an inhibitory theme: Phasic and tonic activation of GABA A receptors. Nat. Rev. Neurosci..

[68] Raghavendra Rao V.L., Dhodda V.K., Song G., Bowen K.K., Dempsey R.J. (2003). Traumatic brain injury-induced acute gene expression changes in rat cerebral cortex identified by GeneChip analysis. J. Neurosci. Res..

[69] Kim G.H., Kang I., Jeong H., Park S., Hong H., Kim J., Kim J., Edden R., Lyoo I.K., Yoon S. (2019). Low prefrontal GABA levels are associated with poor cognitive functions in professional boxers. Front. Hum. Neurosci..

[70] Yasen A.L., Smith J., Christie A.D. (2018). Glutamate and GABA concentrations following mild traumatic brain injury: A pilot study. J. Neurophysiol..

[71] De Beaumont L., Tremblay S., Poirier J., Lassonde M., Théoret H. (2011). Altered bidirectional plasticity and reduced implicit motor learning in concussed athletes. Cereb. Cortex.

[72] De Beaumont L., Lassonde M., Leclerc S., Théoret H. (2007). Long-term and cumulative effects of sports concussion on motor cortex inhibition. Neurosurgery.

[73] Hossain I., Tan C., Dutta G., Siddiqui S., Iasemidis L.D., Arumugam P.U. (2018). A novel electrochemical microbiosensor microarray for real-time continuous in vitro monitoring of gamma-aminobutyric acid. Front. Neurosci..

[74] Burmeister J.J., Price D.A., Pomerleau F., Huettl P., Quintero J.E., Gerhardt G.A. (2020). Challenges of simultaneous measurements of brain extracellular GABA and glutamate in vivo using enzyme-coated microelectrode arrays. J. Neurosci. Methods.

[75] Zhang L., Yang K.H., King A.I. (2001). Comparison of brain responses between frontal and lateral impacts by finite element modeling. J. Neurotrauma.

[76] Horn A.S. (1990). Dopamine uptake: A review of progress in the last decade. Prog. Neurobiol..

[77] Kraus M.F., Smith G.S., Butters M., Donnell A.J., Dixon E., Yilong C., Marion D. (2005). Effects of the dopaminergic agent and NMDA receptor antagonist amantadine on cognitive function, cerebral glucose metabolism and D2 receptor availability in chronic traumatic brain injury: A study using positron emission tomography (PET). Brain Inj..

[78] Wagner A.K., Scanion J.M., Becker C.R., Ritter A.C., Niyonkuru C., Dixon C.E., Conley Y.P., Price J.C. (2014). The influence of genetic variants on striatal dopamine transporter and D2 receptor binding after TBI. J. Cereb. Blood Flow Metab..

[79] Donnemiller E., Brenneis C., Wissel J., Scherfler C., Poewe W., Riccabona G., Wenning G.K. (2000). Impaired dopaminergic neurotransmission in patients with traumatic brain injury: A SPET study using 123 I-β-CIT and 123 I-IBZM. Eur. J. Nucl. Med..

[80] Hastings T.G. (1995). Enzymatic oxidation of dopamine: The role of prostaglandin H synthase. J. Neurochem..

[81] Jenkins P.O., De Simoni S., Bourke N.J., Fleminger J., Scott G., Towey D.J., Svensson W., Khan S., Patel M., Greenwood R. (2018). Dopaminergic abnormalities following traumatic brain injury. Brain.

[82] Van Bregt D.R., Thomas T.C., Hinzman J.M., Cao T., Liu M., Bing G., Gerhardt G.A., Pauly J.R., Lifshitz J. (2012). Substantia nigra vulnerability after a single moderate diffuse brain injury in the rat. Exp. Neurol..

[83] Reeves T.M., Phillips L.L., Povlishock J.T. (2005). Myelinated and unmyelinated axons of the corpus callosum differ in vulnerability and functional recovery following traumatic brain injury. Exp. Neurol..

[84] Massucci J.L., Kline A.E., Ma X., Zafonte R.D., Dixon C.E. (2004). Time dependent alterations in dopamine tissue levels and metabolism after experimental traumatic brain injury in rats. Neurosci. Lett..

[85] Kobori N., Clifton G.L., Dash P.K. (2006). Enhanced catecholamine synthesis in the prefrontal cortex after traumatic brain injury: Implications for prefrontal dysfunction. J. Neurotrauma.

[86] Wagner A.K., Sokoloski J.E., Ren D., Chen X., Khan A.S., Zafonte R.D., Michael A.C., Dixon C.E. (2005). Controlled cortical impact injury affects dopaminergic transmission in the rat striatum. J. Neurochem..

[87] Wagner A.K., Drewencki L.L., Chen X., Santos F.R., Khan A.S., Harun R., Torres G.E., Michael A.C., Dixon C.E. (2009). Chronic methylphenidate treatment enhances striatal dopamine neurotransmission after experimental traumatic brain injury. J. Neurochem..

[88] Liu M., Bachstetter A.D., Cass W.A., Lifshitz J., Bing G. (2017). Pioglitazone attenuates neuroinflammation and promotes dopaminergic neuronal survival in the nigrostriatal system of rats after diffuse brain injury. J. Neurotrauma.

[89] Tang Y.P., Noda Y., Nabeshima T. (1997). Involvement of activation of dopamineraic neuronal system in learning and memory deficits associated with experimental mild traumatic brain injury. Eur. J. Neurosci..

[90] Arciniegas D.B., Held K., Wagner P. (2002). Cognitive impairment following traumatic brain injury. Curr. Treat. Options Neurol..

[91] Bales J.W., Wagner A.K., Kline A.E., Dixon C.E. (2009). Persistent cognitive dysfunction after traumatic brain injury: A dopamine hypothesis. Neurosci. Biobehav. Rev..

[92] Cools R., D’Esposito M. (2011). Inverted-U–shaped dopamine actions on human working memory and cognitive control. Biol. Psychiatry.

[93] Boyeson M.G., Feeney D.M. (1990). Intraventricular norepinephrine facilitates motor recovery following sensorimotor cortex injury. Pharmacol. Biochem. Behav..

[94] Goldstein L.B., Coviello A., Miller G.D., Davis J.N. (1991). Norepinephrine depletion impairs motor recovery following sensorimotor cortex injury in the rat. Restor. Neurol. Neurosci..

[95] McIntosh T.K., Yu T., Gennarelli T.A. (1994). Alterations in regional brain catecholamine concentrations after experimental brain injury in the rat. J. Neurochem..

[96] Dunn-Meynell A., Shijun P., Levin B.E. (1994). Focal traumatic brain injury causes widespread reductions in rat brain norepinephrine turnover from 6 to 24 h. Brain Res..

[97] Dunn-Meynell A.A., Hassanain M., Levin B.E. (1998). Norepinephrine and traumatic brain injury: A possible role in post-traumatic edema. Brain Res..

[98] Levin B.E., Brown K.L., Pawar G., Dunn-Meynell A. (1995). Widespread and lateralized effects of acute traumatic brain injury on norepinephrine turnover in the rat brain. Brain Res..

[99] Fujinaka T., Kohmura E., Yuguchi T., Yoshimine T. (2003). The morphological and neurochemical effects of diffuse brain injury on rat central noradrenergic system. Neurol. Res..

[100] Ramos B.P., Arnsten A.F. (2007). Adrenergic pharmacology and cognition: Focus on the prefrontal cortex. Pharmacol. Ther..

[101] Arnsten A.F., Li B.M. (2005). Neurobiology of executive functions: Catecholamine influences on prefrontal cortical functions. Biol. Psychiatry.

[102] Bonnelle V., Leech R., Kinnunen K.M., Ham T.E., Beckmann C.F., De Boissezon X., Greenwood R.J., Sharp D.J. (2011). Default mode network connectivity predicts sustained attention deficits after traumatic brain injury. J. Neurosci..

[103] Picciotto M.R., Higley M.J., Mineur Y.S. (2012). Acetylcholine as a neuromodulator: Cholinergic signaling shapes nervous system function and behavior. Neuron.

[104] Arciniegas D., Olincy A., Topkoff J., McRae K., Cawthra E., Filley C.M., Reite M., Adler L.E. (2000). Impaired auditory gating and P50 nonsuppression following traumatic brain injury. J. Neuropsychiatry Clin. Neurosci..

[105] Tower D.B., McEachern D. (1948). Acetylcholine and neuronal activity in craniocerebral trauma. J. Clin. Investig..

[106] Tower D.B., McEachern D. (1949). Acetylcholine and neuronal activity: I. Cholinesterase patterns and acetylcholine in the cerebrospinal fluids of patients with craniocerebral trauma. Can. J. Res..

[107] Arciniegas D.B. (2011). Cholinergic dysfunction and cognitive impairment after traumatic brain injury. Part 2: Evidence from basic and clinical investigations. J. Head Trauma Rehabil..

[108] Sachs E. (1957). Acetylcholine and serotonin in the spinal fluid. J. Neurosurg..

[109] Lyeth B.G., Jiang J.Y., Robinson S.E., Guo H., Jenkins L.W. (1993). Hypothermia blunts acetylcholine increase in CSF of traumatically brain injured rats. Mol. Chem. Neuropathol..

[110] Dewar D., Graham D.I. (1996). Depletion of choline acetyltransferase activity but preservation of Ml and M2 muscarinic receptor binding sites in temporal cortex following head injury: A preliminary human postmortem study. J. Neurotrauma.

[111] Salmond C.H., Chatfield D.A., Menon D.K., Pickard J.D., Sahakian B.J. (2004). Cognitive sequelae of head injury: Involvement of basal forebrain and associated structures. Brain.

[112] Zhang L., Plotkin R.C., Wang G., Sandel M.E., Lee S. (2004). Cholinergic augmentation with donepezil enhances recovery in short-term memory and sustained attention after traumatic brain injury. Arch. Phys. Med. Rehabil..

[113] Becker R.E., Kapogiannis D., Greig N.H. (2018). Does traumatic brain injury hold the key to the Alzheimer’s disease puzzle?. Alzheimers Dement..

[114] H. Ferreira-Vieira T., M. Guimaraes I., R. Silva F., M. Ribeiro F. (2016). Alzheimer’s disease: Targeting the cholinergic system. Curr. Neuropharmacol..

[115] Gorman L.K., Fu K., Hovda D.A., Becker D.P., Katayama Y. (1989). Analysis of acetylcholine release following concussive brain injury in the rat. J. Neurotrauma.

[116] Saija A., Hayes R.L., Lyeth B.G., Dixon C.E., Yamamoto T., Robinson S.E. (1988). The effect of concussive head injury on central cholinergic neurons. Brain Res..

[117] Arciniegas D.B. (2003). The cholinergic hypothesis of cognitive impairment caused by traumatic brain injury. Curr. Psychiatry Rep..

[118] Everitt B.J., Robbins T.W. (1997). Central cholinergic systems and cognition. Annu. Rev. Psychol..

[119] Gorman L.K., Fu K., Hovda D.A., Murray M., Traystman R.J. (1996). Effects of traumatic brain injury on the cholinergic system in the rat. J. Neurotrauma.

[120] Dixon C.E., Bao J., Bergmann J.S., Johnson K.M. (1994). Traumatic brain injury reduces hippocampal high-affinity [3H] choline uptake but not extracellular choline levels in rats. Neurosci. Lett..

[121] Dixon C.E., Bao J., Long D.A., Hayes R.L. (1996). Reduced evoked release of acetylcholine in the rodent hippocampus following traumatic brain injury. Pharmacol. Biochem. Behav..

[122] Dixon C.E., Ma X., Marion D.W. (1997). Reduced evoked release of acetylcholine in the rodent neocortex following traumatic brain injury. Brain Res..

[123] Dixon C.E., Bao J., Johnson K.M., Yang K., Whitson J., Clifton G.L., Hayes R.L. (1995). Basal and scopolamine-evoked release of hippocampal acetylcholine following traumatic brain injury in rats. Neurosci. Lett..

[124] Leonard J.R., Maris D.O., Grady M.S. (1994). Fluid percussion injury causes loss of forebrain choline acetyltransferase and nerve growth factor receptor immunoreactive cells in the rat. J. Neurotrauma.

[125] Ciallella J.R., Yan H.Q., Ma X., Wolfson B.M., Marion D.W., DeKosky S.T., Dixon C.E. (1998). Chronic effects of traumatic brain injury on hippocampal vesicular acetylcholine transporter and M2 muscarinic receptor protein in rats. Exp. Neurol..

[126] Dixon C.E., Kochanek P.M., Yan H.Q., Schiding J.K., Griffith R.G., Baum E., Marion D.W., DeKosky S.T. (1999). One-year study of spatial memory performance, brain morphology, and cholinergic markers after moderate controlled cortical impact in rats. J. Neurotrauma.

[127] Ruhé H.G., Mason N.S., Schene A.H. (2007). Mood is indirectly related to serotonin, norepinephrine and dopamine levels in humans: A meta-analysis of monoamine depletion studies. Mol. Psychiatry.

[128] Busto R., Dietrich W.D., Globus M.Y.T., Alonso O., Ginsberg M.D. (1997). Extracellular release of serotonin following fluid-percussion brain injury in rats. J. Neurotrauma.

[129] Abe K., Shimada R., Okada Y., Kibayashi K. (2016). Traumatic brain injury decreases serotonin transporter expression in the rat cerebrum. Neurol. Res..

[130] Zhang Z., Rasmussen L., Saraswati M., Koehler R.C., Robertson C., Kannan S. (2018). Traumatic injury leads to inflammation and altered tryptophan metabolism in the juvenile rabbit brain. J. Neurotrauma.

[131] Barnes N.M., Sharp T. (1999). A review of central 5-HT receptors and their function. Neuropharmacology.

[132] Kreutzer J.S., Seel R.T., Gourley E. (2001). The prevalence and symptom rates of depression after traumatic brain injury: A comprehensive examination. Brain Inj..

[133] Yue J., Burke J., Upadhyayula P., Winkler E., Deng H., Robinson C., Pirracchio R., Suen C., Sharma S., Ferguson A. (2017). Selective serotonin reuptake inhibitors for treating neurocognitive and neuropsychiatric disorders following traumatic brain injury: An evaluation of current evidence. Brain Sci..

[134] Gardner R.C., Yaffe K. (2015). Epidemiology of mild traumatic brain injury and neurodegenerative disease. Mol. Cell. Neurosci..

[135] Sweeney M.D., Sagare A.P., Zlokovic B.V. (2018). Blood–brain barrier breakdown in Alzheimer disease and other neurodegenerative disorders. Nat. Rev. Neurol..

[136] Nguyen Q.T., Schroeder L.F., Mank M., Muller A., Taylor P., Griesbeck O., Kleinfeld D. (2010). An in vivo biosensor for neurotransmitter release and in situ receptor activity. Nat. Neurosci..

[137] Watson C.J., Venton B.J., Kennedy R.T. (2006). In vivo measurements of neurotransmitters by microdialysis sampling. Anal. Chem..

[138] Heien M.L., Johnson M.A., Wightman R.M. (2004). Resolving neurotransmitters detected by fast-scan cyclic voltammetry. Anal. Chem..

[139] Khan A.S., Michael A.C. (2003). Invasive consequences of using micro-electrodes and microdialysis probes in the brain. TrAC Trends Anal. Chem..

[140] Hascup K.N., Hascup E.R., Littrell O.M., Hinzman J.M., Werner C.E., Davis V.A., Burmeister J.J., Pomerleau F., Quintero J.E., Huettl P. (2013). Microelectrode array fabrication and optimization for selective neurochemical detection. Microelectrode Biosensors.

[141] Wall M.J., Usowicz M.M. (1997). Development of action potential-dependent and independent spontaneous GABAA receptor-mediated currents in granule cells of postnatal rat cerebellum. Eur. J. Neurosci..

[142] Kullmann D.M., Asztely F. (1998). Extrasynaptic glutamate spillover in the hippocampus: Evidence and implications. Trends Neurosci..

[143] Dunwiddie T.V., Diao L. (1999). Regulation of extracellular adenosine in rat hippocampal slices is temperature dependent: Role of adenosine transporters. Neuroscience.

[144] Diamond M.E., Arabzadeh E. (2013). Whisker sensory system–from receptor to decision. Prog. Neurobiol..

[145] Bramlett H.M., Dietrich W.D. (2015). Long-term consequences of traumatic brain injury: Current status of potential mechanisms of injury and neurological outcomes. J. Neurotrauma.

[146] Simons D.J., Carvell G.E. (1989). Thalamocortical response transformation in the rat vibrissa/barrel system. J. Neurophysiol..

[147] Urbain N., Deschênes M. (2007). Motor cortex gates vibrissal responses in a thalamocortical projection pathway. Neuron.

[148] Hall R.D., Lindholm E.P. (1974). Organization of motor and somatosensory neocortex in the albino rat. Brain Res..

[149] Welker C. (1976). Receptive fields of barrels in the somatosensory neocortex of the rat. J. Comp. Neurol..

[150] Castro-Alamancos M.A., Connors B.W. (1997). Thalamocortical synapses. Prog. Neurobiol..

[151] Lo F.S., Erzurumlu R.S. (2016). Neonatal sensory nerve injury-induced synaptic plasticity in the trigeminal principal sensory nucleus. Exp. Neurol..

[152] Martini F.J., Moreno-Juan V., Filipchuk A., Valdeolmillos M., Lopez-Bendito G. (2018). Impact of thalamocortical input on barrel cortex development. Neuroscience.

[153] Sofroniew N.J., Vlasov Y.A., Hires S.A., Freeman J., Svoboda K. (2015). Neural coding in barrel cortex during whisker-guided locomotion. eLife.

[154] McNamara K.C., Lisembee A.M., Lifshitz J. (2010). The whisker nuisance task identifies a late-onset, persistent sensory sensitivity in diffuse brain-injured rats. J. Neurotrauma.

[155] Lifshitz J., Lisembee A.M. (2012). Neurodegeneration in the somatosensory cortex after experimental diffuse brain injury. Brain Struct. Funct..

[156] Lafrenaye A.D., Krahe T.E., Povlishock J.T. (2014). Moderately elevated intracranial pressure after diffuse traumatic brain injury is associated with exacerbated neuronal pathology and behavioral morbidity in the rat. J. Cereb. Blood Flow Metab..

[157] Lafrenaye A.D., McGinn M.J., Povlishock J.T. (2012). Increased intracranial pressure after diffuse traumatic brain injury exacerbates neuronal somatic membrane poration but not axonal injury: Evidence for primary intracranial pressure-induced neuronal perturbation. J. Cereb. Blood Flow Metab..

[158] Alwis D.S., Yan E.B., Morganti-Kossmann M.C., Rajan R. (2012). Sensory cortex underpinnings of traumatic brain injury deficits. PLoS ONE.

[159] Bhalala O.G., Rahman M.M., Sun L. Whisker Nuisance Test: A Valuable Tool to Assess Tactile Hypersensitivity in Mice. https://bio-protocol.org/e3331.

[160] Bohnen N., Twijnstra A., Wijnen G., Jolles J. (1991). Tolerance for light and sound of patients with persistent post-concussional symptoms 6 months after mild head injury. J. Neurol..

[161] Stern C.D. (2016). Photophobia, light, and color in acquired brain injury. Multidiscip. Care Patient Brain Inj..

[162] Hinzman J.M., Thomas T.C., Burmeister J.J., Quintero J.E., Huettl P., Pomerleau F., Gerhardt G.A., Lifshitz J. (2010). Diffuse brain injury elevates tonic glutamate levels and potassium-evoked glutamate release in discrete brain regions at two days post-injury: An enzyme-based microelectrode array study. J. Neurotrauma.

[163] Hinzman J.M., Thomas T.C., Quintero J.E., Gerhardt G.A., Lifshitz J. (2012). Disruptions in the regulation of extracellular glutamate by neurons and glia in the rat striatum two days after diffuse brain injury. J. Neurotrauma.

[164] Rutherford E.C., Pomerleau F., Huettl P., Strömberg I., Gerhardt G.A. (2007). Chronic second-by-second measures of l-glutamate in the central nervous system of freely moving rats. J. Neurochem..

[165] Hogan B.L., Lunte S.M., Stobaugh J.F., Lunte C.E. (1994). Online coupling of in vivo microdialysis sampling with capillary electrophoresis. Anal. Chem..

[166] Kennedy R.T. (2013). Emerging trends in in vivo neurochemical monitoring by microdialysis. Curr. Opin. Chem. Biol..

[167] Hascup E.R., Hascup K.N., Stephens M., Pomerleau F., Huettl P., Gratton A., Gerhardt G.A. (2010). Rapid microelectrode measurements and the origin and regulation of extracellular glutamate in rat prefrontal cortex. J. Neurochem..

[168] Chefer V.I., Thompson A.C., Zapata A., Shippenberg T.S. (2009). Overview of brain microdialysis. Curr. Protoc. Neurosci..

[169] Marvin J.S., Borghuis B.G., Tian L., Cichon J., Harnett M.T., Akerboom J., Gordus A., Renninger S.L., Chen T.-W., Bargmann C.I. (2013). An optimized fluorescent probe for visualizing glutamate neurotransmission. Nat. Methods.

[170] Hefendehl J.K., LeDue J., Ko R.W.Y., Mahler J., Murphy T.H., MacVicar B.A. (2016). Mapping synaptic glutamate transporter dysfunction in vivo to regions surrounding Aβ plaques by iGluSnFR two-photon imaging. Nat. Commun..

[171] Bosche B., Dohmen C., Graf R., Neveling M., Staub F., Kracht L., Sobesky J., Lehnhardt F.G., Heiss W.D. (2003). Extracellular concentrations of non–transmitter amino acids in peri-infarct tissue of patients predict malignant middle cerebral artery infarction. Stroke.

[172] Burmeister J.J., Moxon K., Gerhardt G.A. (2000). Ceramic-based multisite microelectrodes for electrochemical recordings. Anal. Chem..

[173] Yang H., Peters J.L., Michael A.C. (1998). Coupled effects of mass transfer and uptake kinetics on in vivo microdialysis of dopamine. J. Neurochem..

[174] Weltin A., Kieninger J., Urban G.A. (2016). Microfabricated, amperometric, enzyme-based biosensors for in vivo applications. Anal. Bioanal. Chem..

[175] Burmeister J.J., Pomerleau F., Palmer M., Day B.K., Huettl P., Gerhardt G.A. (2002). Improved ceramic-based multisite microelectrode for rapid measurements of L-glutamate in the CNS. J. Neurosci. Methods.

[176] Yu P., He X., Zhang L., Mao L. (2014). Dual recognition unit strategy improves the specificity of the adenosine triphosphate (ATP) aptamer biosensor for cerebral ATP assay. Anal. Chem..

[177] Ledo A., Lourenço C.F., Laranjinha J., Brett C.M., Gerhardt G.A., Barbosa R.M. (2017). Ceramic-based multisite platinum microelectrode arrays: Morphological characteristics and electrochemical performance for extracellular oxygen measurements in brain tissue. Anal. Chem..

[178] Hascup E.R., af Bjerkén S., Hascup K.N., Pomerleau F., Huettl P., Strömberg I., Gerhardt G.A. (2009). Histological studies of the effects of chronic implantation of ceramic-based microelectrode arrays and microdialysis probes in rat prefrontal cortex. Brain Res..

[179] Hascup K.N., Hascup E.R. (2014). Electrochemical techniques for subsecond neurotransmitter detection in live rodents. Comp. Med..

[180] Hinzman J.M., Gibson J.L., Tackla R.D., Costello M.S., Burmeister J.J., Quintero J.E., Gerhardt G.A., Hartings J.A. (2015). Real-time monitoring of extracellular adenosine using enzyme-linked microelectrode arrays. Biosens. Bioelectron..

[181] Burmeister J.J., Pomerleau F., Huettl P., Gash C.R., Werner C.E., Bruno J.P., Gerhardt G.A. (2008). Ceramic-based multisite microelectrode arrays for simultaneous measures of choline and acetylcholine in CNS. Biosens. Bioelectron..

[182] Burmeister J.J., Palmer M., Gerhardt G.A. (2005). L-lactate measures in brain tissue with ceramic-based multisite microelectrodes. Biosens. Bioelectron..

[183] Lourenço C.F., Ledo A., Gerhardt G.A., Laranjinha J., Barbosa R.M. (2017). Neurometabolic and electrophysiological changes during cortical spreading depolarization: Multimodal approach based on a lactate-glucose dual microbiosensor arrays. Sci. Rep..

[184] Hall S.B., Khudaish E.A., Hart A.L. (1998). Electrochemical oxidation of hydrogen peroxide at platinum electrodes. Part 1. An adsorption-controlled mechanism. Electrochim. Acta.

[185] Migneault I., Dartiguenave C., Bertrand M.J., Waldron K.C. (2004). Glutaraldehyde: Behavior in aqueous solution, reaction with proteins, and application to enzyme crosslinking. Biotechniques.

[186] Hunsberger H.C., Setti S.E., Heslin R.T., Quintero J.E., Gerhardt G.A., Reed M.N. (2017). Using enzyme-based biosensors to measure tonic and phasic glutamate in Alzheimer’s mouse models. J. Vis. Exp..

[187] Matveeva E.A., Davis V.A., Whiteheart S.W., Vanaman T.C., Gerhardt G.A., Slevin J.T. (2012). Kindling-induced asymmetric accumulation of hippocampal 7S SNARE complexes correlates with enhanced glutamate release. Epilepsia.

[188] Hascup E.R., Hascup K.N., Pomerleau F., Huettl P., Hajos-Korcsok E., Kehr J., Gerhardt G.A. (2012). An allosteric modulator of metabotropic glutamate receptors (mGluR2),(+)-TFMPIP, inhibits restraint stress-induced phasic glutamate release in rat prefrontal cortex. J. Neurochem..

[189] Stephens M.L., Quintero J.E., Pomerleau F., Huettl P., Gerhardt G.A. (2011). Age-related changes in glutamate release in the CA3 and dentate gyrus of the rat hippocampus. Neurobiol. Aging.

[190] Thomas T.C., Beitchman J.A., Pomerleau F., Noel T., Jungsuwadee P., Butterfield D.A., Clair D.K.S., Vore M., Gerhardt G.A. (2017). Acute treatment with doxorubicin affects glutamate neurotransmission in the mouse frontal cortex and hippocampus. Brain Res..

[191] Hascup K.N., Hascup E.R., Pomerleau F., Huettl P., Gerhardt G.A. (2008). Second-by-second measures of L-glutamate in the prefrontal cortex and striatum of freely moving mice. J. Pharmacol. Exp. Ther..

[192] Sabeti J., Gerhardt G.A., Zahniser N.R. (2002). Acute cocaine differentially alters accumbens and striatal dopamine clearance in low and high cocaine locomotor responders: Behavioral and electrochemical recordings in freely moving rats. J. Pharmacol. Exp. Ther..

[193] Wassum K.M., Tolosa V.M., Tseng T.C., Balleine B.W., Monbouquette H.G., Maidment N.T. (2012). Transient extracellular glutamate events in the basolateral amygdala track reward-seeking actions. J. Neurosci..

[194] Dash M.B., Douglas C.L., Vyazovskiy V.V., Cirelli C., Tononi G. (2009). Long-term homeostasis of extracellular glutamate in the rat cerebral cortex across sleep and waking states. J. Neurosci..

[195] Miller E.M., Quintero J.E., Pomerleau F., Huettl P., Gerhardt G.A., Glaser P.E. (2019). Chronic methylphenidate alters tonic and phasic glutamate signaling in the frontal cortex of a freely-moving rat model of ADHD. Neurochem. Res..

[196] Zhang H., Lin S.C., Nicolelis M.A. (2009). Acquiring local field potential information from amperometric neurochemical recordings. J. Neurosci. Methods.

[197] Burmeister J.J., Pomerleau F., Quintero J.E., Huettl P., Ai Y., Jakobsson J., Lundblad M., Heuer A., Slevin J.T., Gerhardt G.A. (2018). In vivo electrochemical studies of optogenetic control of glutamate signaling measured using enzyme-based ceramic microelectrode arrays. Biochemical Approaches for Glutamatergic Neurotransmission.

[198] Disney A.A., McKinney C., Grissom L., Lu X., Reynolds J.H. (2015). A multi-site array for combined local electrochemistry and electrophysiology in the non-human primate brain. J. Neurosci. Methods.

[199] Ledo A., Lourenco C.F., Laranjinha J., Gerhardt G.A., Barbosa R.M. (2018). Concurrent measurements of neurochemical and electrophysiological activity with microelectrode arrays: New perspectives for constant potential amperometry. Curr. Opin. Electrochem..

[200] Aldrin-Kirk P., Heuer A., Wang G., Mattsson B., Lundblad M., Parmar M., Björklund T. (2016). DREADD modulation of transplanted DA neurons reveals a novel parkinsonian dyskinesia mechanism mediated by the serotonin 5-HT6 receptor. Neuron.

[201] Butler C.R., Boychuk J.A., Pomerleau F., Alcala R., Huettl P., Ai Y., Jakobsson J., Whiteheart S.W., Gerhardt G.A., Smith B.N. (2019). Modulation of epileptogenesis: A paradigm for the integration of enzyme-based microelectrode arrays and optogenetics. Epilepsy Res..

[202] Nemani V.M., Manley G.T. (2004). Brain tissue oxygen monitoring: Physiologic principles and clinical application. Oper. Tech. Neurosurg..

[203] De Georgia M.A. (2015). Brain tissue oxygen monitoring in neurocritical care. J. Intensive Care Med..

[204] Cannestra A.F., Pouratian N., Bookheimer S.Y., Martin N.A., Becker D.P., Toga A.W. (2001). Temporal spatial differences observed by functional MRI and human intraoperative optical imaging. Cereb. Cortex.

[205] Connell E., Krishna G., Hair C., Adelson P.D., Thomas T.C. (2019). A novel approach to measuring real-time circuit dysregulation after diffuse brain injury. J. Neurotrauma..

[206] Deadwyler S.A., Hampson R.E., Song D., Opris I., Gerhardt G.A., Marmarelis V.Z., Berger T.W. (2017). A cognitive prosthesis for memory facilitation by closed-loop functional ensemble stimulation of hippocampal neurons in primate brain. Exp. Neurol..

[207] Stephens M.L., Pomerleau F., Huettl P., Gerhardt G.A., Zhang Z. (2010). Real-time glutamate measurements in the putamen of awake rhesus monkeys using an enzyme-based human microelectrode array prototype. J. Neurosci. Methods.

[208] Geyer E.D., Shetty P.A., Suozzi C.J., Allen D.Z., Benavidez P.P., Liu J., Hollis C.N., Gerhardt G.A., Quintero J.E., Burmeister J.J. (2018). Adaptation of microelectrode array technology for the study of anesthesia-induced neurotoxicity in the intact piglet brain. J. Vis. Exp..

[209] Kasasbeh A., Lee K., Bieber A., Bennet K., Chang S.Y. (2013). Wireless neurochemical monitoring in humans. Stereotact. Funct. Neurosurg..

[210] Ferguson M., Sharma D., Ross D., Zhao F. (2019). A Critical review of microelectrode arrays and strategies for improving neural interfaces. Adv. Healthc. Mater..

[211] Klose M., Feldt-Rasmussen U. (2018). Chronic endocrine consequences of traumatic brain injury—What is the evidence?. Nat. Rev. Endocrinol..

[212] Moreau O.K., Yollin E., Merlen E., Daveluy W., Rousseaux M. (2012). Lasting pituitary hormone deficiency after traumatic brain injury. J. Neurotrauma.

[213] Schneider H.J., Kreitschmann-Andermahr I., Ghigo E., Stalla G.K., Agha A. (2007). Hypothalamopituitary dysfunction following traumatic brain injury and aneurysmal subarachnoid hemorrhage: A systematic review. JAMA.

[214] Griesbach G.S., Hovda D.A., Tio D.L., Taylor A.N. (2011). Heightening of the stress response during the first weeks after a mild traumatic brain injury. Neuroscience.

[215] Rowe R.K., Rumney B.M., May H.G., Permana P., Adelson P.D., Harman S.M., Lifshitz J., Thomas T.C. (2016). Diffuse traumatic brain injury affects chronic corticosterone function in the rat. Endocr. Connect..

[216] Russell A.L., Richardson M.R., Bauman B.M., Hernandez I.M., Saperstein S., Handa R.J., Wu T.J. (2018). Differential responses of the HPA axis to mild blast traumatic brain injury in male and female mice. Endocrinology.

[217] De Kloet E.R., Meijer O.C., de Nicola A.F., de Rijk R.H., Joëls M. (2018). Importance of the brain corticosteroid receptor balance in metaplasticity, cognitive performance and neuro-inflammation. Front. Neuroendocrinol..

[218] Ziebell J.M., Rowe R.K., Muccigrosso M.M., Reddaway J.T., Adelson P.D., Godbout J.P., Lifshitz J. (2017). Aging with a traumatic brain injury: Could behavioral morbidities and endocrine symptoms be influenced by microglial priming?. Brain. Behav. Immun..

[219] Frugier T., Morganti-Kossmann M.C., O’Reilly D., McLean C.A. (2010). In situ detection of inflammatory mediators in post mortem human brain tissue after traumatic injury. J. Neurotrauma.

[220] Cosgrove K.P., Mazure C.M., Staley J.K. (2007). Evolving knowledge of sex differences in brain structure, function, and chemistry. Biol. Psychiatry.

[221] De Vries G.J. (1990). Sex differences in neurotransmitter systems. J. Neuroendocrinol..

[222] Madeira M.D., Lieberman A.R. (1995). Sexual dimorphism in the mammalian limbic system. Prog. Neurobiol..

[223] Duman R.S., Sanacora G., Krystal J.H. (2019). Altered connectivity in depression: GABA and glutamate neurotransmitter deficits and reversal by novel treatments. Neuron.

[224] Wunderle M.K., Hoeger K.M., Wasserman M.E., Bazarian J.J. (2014). Menstrual phase as predictor of outcome after mild traumatic brain injury in women. J. Head Trauma Rehabil..

[225] Yokomaku D., Numakawa T., Numakawa Y., Suzuki S., Matsumoto T., Adachi N., Nishio C., Taguchi T., Hatanaka H. (2003). Estrogen enhances depolarization-induced glutamate release through activation of phosphatidylinositol 3-kinase and mitogen-activated protein kinase in cultured hippocampal neurons. Mol. Endocrinol..

[226] Yankova M., Hart S.A., Woolley C.S. (2001). Estrogen increases synaptic connectivity between single presynaptic inputs and multiple postsynaptic CA1 pyramidal cells: A serial electron-microscopic study. Proc. Natl. Acad. Sci..

[227] Kao C.H., ChangLai S.P., Chieng P.U., Yen T.C. (1998). Gastric emptying in head-injured patients. Am. J. Gastroenterol..

[228] Ott L., Young B., Phillips R., McClain C., Adams L., Dempsey R., Tibbs P., Ryo U.Y. (1991). Altered gastric emptying in the head-injured patient: Relationship to feeding intolerance. J. Neurosurg..

[229] Olsen A.B., Hetz R.A., Xue H., Aroom K.R., Bhattarai D., Johnson E., Bedi S., Cox C.S., Uray K. (2013). Effects of traumatic brain injury on intestinal contractility. Neurogastroenterol. Motil..

[230] Breit S., Kupferberg A., Rogler G., Hasler G. (2018). Vagus nerve as modulator of the brain–gut axis in psychiatric and inflammatory disorders. Front. Psychiatry.

[231] Neren D., Johnson M.D., Legon W., Bachour S.P., Ling G., Divani A.A. (2016). Vagus nerve stimulation and other neuromodulation methods for treatment of traumatic brain injury. Neurocrit. Care.

[232] Cryan J.F., O’Mahony S.M. (2011). The microbiome-gut-brain axis: From bowel to behavior. Neurogastroenterol. Motil..

[233] Treangen T.J., Wagner J., Burns M.P., Villapol S. (2018). Traumatic brain injury in mice induces acute bacterial dysbiosis within the fecal microbiome. Front. Immunol..

[234] Nicholson S.E., Watts L.T., Burmeister D.M., Merrill D., Scroggins S., Zou Y., Lai Z., Grandhi R., Lewis A.M., Newton L.M. (2018). Moderate traumatic brain injury alters the gastrointestinal microbiome in a time-dependent manner. Shock.

[235] Neufeld K.M., Kang N., Bienenstock J., Foster J.A. (2011). Reduced anxiety-like behavior and central neurochemical change in germ-free mice. Neurogastroenterol. Motil..

[236] Lyte M. (2011). Probiotics function mechanistically as delivery vehicles for neuroactive compounds: Microbial endocrinology in the design and use of probiotics. Bioessays.

[237] Cryan J.F., Dinan T.G. (2012). Mind-altering microorganisms: The impact of the gut microbiota on brain and behaviour. Nat. Rev. Neurosci..

[238] Strandwitz P. (2018). Neurotransmitter modulation by the gut microbiota. Brain Res..

[239] Ozogul F., Kuley E., Ozogul Y., Ozogul I. (2012). The function of lactic acid bacteria on biogenic amines production by food-borne pathogens in arginine decarboxylase broth. Food Sci. Technol. Res..

[240] O’Mahony S.M., Clarke G., Borre Y.E., Dinan T.G., Cryan J.F. (2015). Serotonin, tryptophan metabolism and the brain-gut-microbiome axis. Behav. Brain Res..

[241] Barrett E., Ross R.P., O’toole P.W., Fitzgerald G.F., Stanton C. (2012). γ-Aminobutyric acid production by culturable bacteria from the human intestine. J. Appl. Microbiol..

[242] Das M., Mohapatra S., Mohapatra S.S. (2012). New perspectives on central and peripheral immune responses to acute traumatic brain injury. J. Neuroinflammation.

[243] Utagawa A., Truettner J.S., Dietrich W.D., Bramlett H.M. (2008). Systemic inflammation exacerbates behavioral and histopathological consequences of isolated traumatic brain injury in rats. Exp. Neurol..

[244] Villapol S., Kryndushkin D., Balarezo M.G., Campbell A.M., Saavedra J.M., Shewmaker F.P., Symes A.J. (2015). Hepatic expression of serum amyloid A1 is induced by traumatic brain injury and modulated by telmisartan. Am. J. Pathol..

[245] Chen X., Pan Z., Fang Z., Lin W., Wu S., Yang F., Li Y., Fu H., Gao H., Li S. (2018). Omega-3 polyunsaturated fatty acid attenuates traumatic brain injury-induced neuronal apoptosis by inducing autophagy through the upregulation of SIRT1-mediated deacetylation of Beclin-1. J. Neuroinflammation.

[246] Zhao Q., Yan T., Li L., Chopp M., Venkat P., Qian Y., Li R., Wu R., Li W., Lu M. (2019). Immune response mediates cardiac dysfunction after traumatic brain injury. J. Neurotrauma.

[247] Felten S.Y., Olschowka J. (1987). Noradrenergic sympathetic innervation of the spleen: II. Tyrosine hydroxylase (TH)-positive nerve terminals form synaptic like contacts on lymphocytes in the splenic white pulp. J. Neurosci. Res..

[248] Das M., Leonardo C.C., Rangooni S., Pennypacker K.R., Mohapatra S., Mohapatra S.S. (2011). Lateral fluid percussion injury of the brain induces CCL20 inflammatory chemokine expression in rats. J. Neuroinflammation.

[249] Straub R.H. (2004). Complexity of the bi-directional neuroimmune junction in the spleen. Trends Pharmacol. Sci..

